# The Transcription Factor Hif-1 Enhances the Radio-Resistance of Mouse MSCs

**DOI:** 10.3389/fphys.2018.00439

**Published:** 2018-04-26

**Authors:** Irene Calvo-Asensio, Eugène T. Dillon, Noel F. Lowndes, Rhodri Ceredig

**Affiliations:** ^1^Regenerative Medicine Institute, School of Medicine, Nursing and Health Sciences, National University of Ireland, Galway, Ireland; ^2^Genome Stability Laboratory, Centre for Chromosome Biology, National University of Ireland, Galway, Ireland; ^3^Proteome Research Centre, Conway Institute of Biomolecular and Biomedical Research, University College Dublin, Dublin, Ireland

**Keywords:** mesenchymal stromal cells, DNA damage response, ionizing radiation, hypoxia, label-free proteomics

## Abstract

Mesenchymal stromal cells (MSCs) are multipotent progenitors supporting bone marrow hematopoiesis. MSCs have an efficient DNA damage response (DDR) and are consequently relatively radio-resistant cells. Therefore, MSCs are key to hematopoietic reconstitution following total body irradiation (TBI) and bone marrow transplantation (BMT). The bone marrow niche is hypoxic and via the heterodimeric transcription factor Hypoxia-inducible factor-1 (Hif-1), hypoxia enhances the DDR. Using gene knock-down, we have previously shown that the Hif-1α subunit of Hif-1 is involved in mouse MSC radio-resistance, however its exact mechanism of action remains unknown. In order to dissect the involvement of Hif-1α in the DDR, we used CRISPR/Cas9 technology to generate a stable mutant of the mouse MSC cell line MS5 lacking Hif-1α expression. Herein, we show that it is the whole Hif-1 transcription factor, and not only the Hif-1α subunit, that modulates the DDR of mouse MSCs. This effect is dependent upon the presence of a Hif-1α protein capable of binding to both DNA and its heterodimeric partner Arnt (Hif-1β). Detailed transcriptomic and proteomic analysis of *Hif1a* KO MS5 cells leads us to conclude that Hif-1α may be acting indirectly on the DNA repair process. These findings have important implications for the modulation of MSC radio-resistance in the context of BMT and cancer.

## Introduction

Hematopoiesis is the process whereby through a complex series of commitment and differentiation events, hematopoietic stem cells (HSC) differentiate into all types of blood cells (Brown et al., 2015; Kumar and Evans, 2015). In adults, the bone marrow is the primary organ supporting hematopoiesis, and constitutes a highly specialized environment in which both non-hematopoietic stromal cells and hematopoietic cells in various developmental stages form the bone marrow niche (Li and Li, 2006; Lo Celso and Scadden, 2011). Mesenchymal stromal cells (MSCs) are key components of the bone marrow microenvironment, providing support for HSCs and regulating their maintenance and the production and maturation of hematopoietic progenitors (Nagasawa et al., 2011). Apart from providing microenvironmental support for HSCs, MSCs are themselves also multi-potent progenitors capable of differentiating along various mesenchymal lineages to become adipocytes (fat cells), osteocytes (bone cells), and chondrocytes (cartilage cells) (Pittenger et al., 1999). In addition to the bone marrow, MSCs are also known to localize to solid tumors to which they are attracted following signals from the tumor mass and therein develop into cancer-associated fibroblasts (CAFs), becoming an integral part of the tumor microenvironment and promoting tumorigenesis in multiple ways (Mishra et al., 2009; Kraman et al., 2010; Hanahan and Weinberg, 2011; Cuiffo and Karnoub, 2012; Hanahan and Coussens, 2012; Kidd et al., 2012; Barcellos-de-Souza et al., 2013; Poggi et al., 2014). A feature shared by the bone marrow niche and tumors is that they are relatively hypoxic environments (Vaupel, 2004; Ruan et al., 2009; Eliasson and Jönsson, 2010; Mohyeldin et al., 2010; Spencer et al., 2014). Hypoxia has been shown to play a key role in both HSC and MSC biology, modulating their proliferation rates, quiescence, differentiation potential, and re-constitutive capacity (Eliasson and Jönsson, 2010; Guitart et al., 2010; Jin et al., 2010; Mohyeldin et al., 2010; Winkler et al., 2010; Tsai et al., 2012a,b; Prado-Lòpez et al., 2014). In the tumor microenvironment, hypoxia is involved in angiogenesis, proliferation, metabolism, metastasis, differentiation, and response to radiation therapy, therefore being an important adverse prognostic factor in cancer (Hall and Giaccia, 2006; Bristow and Hill, 2008; Rankin and Giaccia, 2008; Philip et al., 2013).

Cellular adaptation to hypoxia is mediated by a family of transcription factors known as hypoxia inducible factors, or HIFs. These consist of a constitutively expressed HIF-1β subunit (also known as ARNT) and an oxygen-responsive HIF-α subunit (HIF-1α, HIF-2α, or HIF-3α) (Mazumdar et al., 2009). Amongst these, HIF-1α is considered to be the main contributor to the general acute cellular response to hypoxia, mainly because it is ubiquitously expressed in all tissues, whereas its HIF-2α and HIF-3α counterparts are expressed in a tissue-specific fashion and are thought to contribute to more specific processes (Wenger et al., 1996; Ema et al., 1997; Tian et al., 1997; Elvert et al., 1999; Semenza, 2000; Makino et al., 2001; Ke and Costa, 2006; Yang et al., 2015). All members of the HIF family share their protein domain structure, which is composed mainly of a DNA-binding bHLH domain and a tandem of two PAS domains (PAS-A and PAS-B) for heterodimerization (Dengler et al., 2014). Apart from these, the alpha subunits also contain an oxygen-dependent degradation domain (ODDD) involved in the regulation of protein stability, and transactivation domains (TAD) for the regulation of protein function (Jiang et al., 1996, 1997; Pugh et al., 1997; Gu et al., 1998; Huang et al., 1998; Hara et al., 2001; Ke and Costa, 2006). In normoxic conditions, proline residues in the HIF-α degradation domain (ODDD) are hydroxylated by prolyl hydroxylase domain-containing proteins (PHDs), enabling the interaction with the von Hippel Lindau tumor suppressor protein pVHL, which ultimately results in its proteasomal degradation (Kamura et al., 2000; Bruick and McKnight, 2001; Ivan and Kaelin, 2001; Masson et al., 2001; Masson and Ratcliffe, 2003; Ke and Costa, 2006; Benizri et al., 2008). In hypoxic conditions, however, these enzymes no longer post-translationally modify HIF-1α, allowing its stabilization and accumulation within the cells. Stabilized HIF-1α is subsequently activated and translocated to the nucleus where it dimerizes with ARNT (HIF-1β), forming the fully-active HIF-1 transcription factor, capable of regulating the expression of hypoxia-responsive genes (Semenza, 2000; Hofer et al., 2002; Ke and Costa, 2006; Lendahl et al., 2009). HIF-1-dependent regulation has been attributed to a myriad of genes involving many important cellular processes such as the metabolic switch to glycolysis, angiogenesis, growth factor and cytokine production, cell growth and migration, extracellular matrix production, autophagy, apoptosis, redox homeostasis, inflammation and immunity, and the DNA damage response (DDR), all of them crucial for cells to functionally adapt to low oxygen environments (Ke and Costa, 2006; Xia and Kung, 2009; Semenza, 2012; Rohwer et al., 2013; Dengler et al., 2014; Wu et al., 2015). However, HIF-1 activity in these biological processes is often tissue- or cell-type specific due to the impact of the particular chromatin status, RNA Pol II activity, and the availability of partner transcription factors or co-activators found in each cell type (Xia and Kung, 2009; Pawlus et al., 2012, 2014; Villar et al., 2012; Galbraith et al., 2013; Pawlus and Hu, 2013).

Previous studies by our group comparing how MSC respond to ionizing radiation in normoxia (21% O_2_) and hypoxia (5–2% O_2_) have shown that *in vitro*, both primary mouse MSCs and MSC lines display a hypoxia-dependent increase in resistance to γ-irradiation (Sugrue et al., 2014). In the nuclei of irradiated MSC, DNA lesions were resolved at a faster rate in hypoxia. In addition, hypoxia enhanced the ability of MSC to repair DNA lesions by both non-homologous end joining (NHEJ) and homologous repair (HR) mechanisms. Interestingly, upon exposure of MSC to hypoxia, the levels of the key NHEJ repair proteins DNA-PKcs and DNA ligase IV (but not the HR factor Rad51) increased coinciding with Hif-1α stabilization and accumulation in the cells, and this increase was prevented by small interfering RNA (siRNA)-mediated depletion of Hif-1α (Sugrue et al., 2014).

Here we have studied the molecular mechanism underlying the role of Hif-1α in the DDR of hypoxic MSCs. Our results indicate that both subunits of the Hif-1 transcription factor, and their interaction, are essential for mediating the hypoxia-dependent increase in MSC radio-resistance. In addition, by using site-directed mutagenesis, we show that conservation of the amino acids responsible for the direct interaction of Hif-1α with the DNA are also crucial for this effect, indicating that it might be doing so thanks to its transcription factor function. However, the fact that hypoxia did not cause changes in mRNA expression of almost any of the 87 DDR genes analyzed, especially DNA-PK_cs_, and DNA ligase IV, whose protein levels are increased by exposure to hypoxia in a Hif-1α-dependent manner, is an important indicator that Hif-1 may be indirectly affecting DDR protein stability through the transcriptional regulation of an (or several) intermediary factor(s).

## Materials and methods

### Cell culture and treatments

The mouse MSC cell line MS5 was originally provided by Prof. Antonius Rolink (Department of Biomedicine, University of Basel) and was cultured in Dulbecco's modified Eagle's medium high glucose (Gibco) supplemented with 10% fetal bovine serum (FBS) (Sigma Aldrich) and 1% penicillin/streptomycin sulfate solution (Gibco). Recloned MS5 cells were continuously cultured in humidified incubators at 37°C containing 21% O_2_ (normoxia) or 2% O_2_ (hypoxia) for at least 1 week prior to experimentation, and were routinely screened for mycoplasma contamination. Previous investigations in our group (Sugrue et al., 2014) were performed using oxygen concentrations ranging from 5 to 2% O_2_, and no differences in the results obtained were observed between the two oxygen concentrations. For this reason, the oxygen level chosen for our studies was 2% O_2_. Experiments that required hypoxia treatment were performed in a hypoxic chamber (Coy Lab Products). Cells were γ-irradiated at the indicated doses using a Mainance Millennium Sample Irradiator containing a ^137^Cs source at a dose rate of approximately 102 cGy/min. When applicable, cells were treated with 500 μM Dimethyloxalylglycine (DMOG) (Sigma Aldrich) and harvested at the indicated time points post-treatment. siRNA transfection was performed using Oligofectamine reagent (Invitrogen). Mouse Arnt Silencer® Select Predesigned siRNA (s62616) and Silencer® Select Negative Control No. 1 siRNA (4390843) were obtained from Ambion. 1.5 × 10^5^ MS5 cells were seeded per 60 mm tissue culture plate. 24 h later, cells were transfected with 50 nM of siRNA using 6 μl of Oligofectamine (Invitrogen) in 1 ml of Opti-MEM I (Invitrogen) per 60 mm dish. 3 h post transfection, 0.5 ml of DMEM (without penicillin and streptomycin) supplemented with 30% FBS and 4 mM L-glutamine was added. After 24 h, medium was substituted with the usual DMEM with 10% FBS and 1% penicillin-streptomycin. 48 h after transfection, knockdown efficiency was assessed by western blotting and cells were used for the required experiments.

### Clonogenic survival assay

Control unirradiated or MS5 cells irradiated with 2–10Gy were seeded into six-well plates (Nunc) at a concentration of 300 cells per well. Cells were incubated for 8 days until colonies were clearly visible. Colonies were stained with Coomassie Blue (Sigma-Aldrich) and counted. The percentage survival of each cell type was determined by normalizing the number of colonies per cell generated by irradiated cultures to the number of colonies per cell generated by control cultures.

### Plasmid and cell line construction

Details about the generation of all plasmids and cell lines used for these studies, including the CRISPR/Cas9 methodology used to disrupt Hif-1α from the MS5 mouse MSC cell line, can be found in the Supplementary Materials.

### Western blotting

Whole cell extracts were prepared from control or irradiated cells at the indicated time-points post irradiation by direct addition of 1 × Laemmli buffer to cells that still adhered to the culture plates following one wash with ice-cold PBS. Cells were disaggregated into the Laemmli buffer using a cell scraper, heated at 95°C for 5 min, sonicated and spun down at 14,000 g for 2 min at 4°C prior to quantifying protein content by the Bradford method. 30–50 μg of total cell extracts were separated using SDS-PAGE gels and transferred to nitrocellulose membranes. Chemiluminescence was detected using SuperSignal West Pico Chemiluminescent Substrate (Thermo Scientific) and medical x-ray film (Konica Minolta Medical & Graphic Imaging Inc.,). In assays in which protein quantification was necessary, this was performed using a LiCor Odissey infrared imaging system according to manufacturer's instructions.

### qPCR

Total RNA was isolated from cells by TRIzol® Reagent (Life Technologies). cDNA was generated using Applied Biosystem's High-Capacity cDNA Reverse Transcription Kit according to the manufacturer's instructions. The resulting cDNA was used as a template in quantitative PCR reactions with specific primers on a Step One Plus Real-Time PCR System (Applied Biosystems). The reactions were prepared with SYBR Select reaction mix from Applied Biosystems. Predesigned KiCqStart® primer pairs for mouse DNA-PKcs (*Prkdc)*, DNA ligase IV *(Lig4), Rad51*, 53BP1 *(Tp53bp1), Mdc1, Brca1, AldoA, Bnip3, Egln1*, and β-Actin (*Actb*) were purchased from Sigma Aldrich. Gene expression analysis was carried out using the 2^−ΔΔCt^ method and β-Actin was used as control gene for normalization. Results were adjusted according to the primer efficiencies previously calculated.

### DNA damage response qPCR arrays

RNA was isolated from MS5 cells cultured in normoxia (21% O_2_) or hypoxia (2% O_2_) using the TRIzol-chloroform method. 500 ng per sample of the resulting total RNA were used as a template for cDNA synthesis using Quiagen's RT2 First Strand Kit according to the manufacturer's protocol. qPCR reactions were prepared using the RT2 SYBR Green ROX qPCR Mastermix from Quiagen and loaded into the commercial customized Mouse DDR RT2 Profiler PCR Arrays which include primers for DNA Ligase IV (*Lig4*), *Bcl2*, Bcl-XL (*Bcl2l1*), and Puma (*Bbc3*) in addition to the 84 DDR genes present in the standard PCR arrays.

### Label-free proteomic analysis

WT and *Hif1a*^−/−^ MS5 cells were cultured in normoxia (21% O_2_) or hypoxia (2% O_2_) for 7 days before harvesting for protein extraction. For this, cells were washed in ice-cold PBS and lysed in 8 M urea, sonicated and spun down at 14,000 g for 2 min at 4°C to remove aggregates. Protein was then purified and processed as described in the Supplementary Materials. The mass spectrometry proteomics data have been deposited to the ProteomeXchange Consortium via the PRIDE (Vizcaíno et al., 2016) partner repository with the dataset identifier PXD006701.

## Results

### Hif-1α-mediated increase of the radio-resistance of hypoxic mouse MS5 cells is not caused by an effect on the mRNA expression of DDR factors

**Figure 1:**
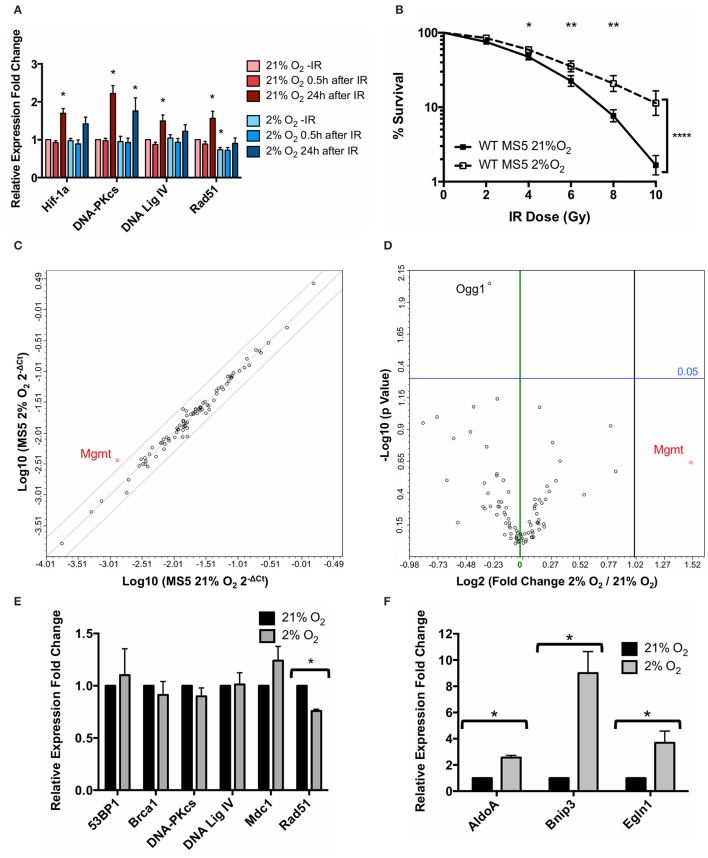
Hypoxia treatment does not affect mRNA expression of DNA Damage Response genes. **(A)** mRNA expression levels of *Hif1a* and the DDR factors DNA-PKcs (*Prkdc*), DNA Ligase IV (*Lig4*), and *Rad51*, measured 0–24 h after treatment with 10Gy IR. β-actin (*Actb*) was used as housekeeping gene and normoxic samples as reference for mRNA relative expression quantification using the ΔΔCt method. ^*^p < 0.05, paired *t*-tests, *n* = 9. **(B)** Clonogenic survival assay of MS5 cells in 21 or 2% O_2_. ^*^p < 0.05, ^**^p < 0.01, ^****^p < 0.0001, compared with normoxic samples, two-way ANOVA with Bonferroni post-test correction for the multiple comparisons, *n* = 9. **(C)** Scatter plot and **(D)** volcano plot of qPCR array data comparing normoxic (21% O_2_) and hypoxic (2% O_2_) MS5 cells. In the scatter plot, diagonal lines represent − 2, 0, and +2-fold changes, from left to right. In the volcano plot, green and black vertical lines represent 0 and 2-fold expression changes, respectively. Blue horizontal lines represent a p value of 0.05 (*n* = 3), with significantly regulated genes being shown above them. All genes up-regulated more than 2-fold are shown in red (independently of their statistical significance). mRNA expression levels of **(E)** DDR factors 53BP1 (*Tp53bp1*), *Brca1*, DNA-PKcs (*Prkdc*), DNA Ligase IV (*Lig4*), *Mdc1* and *Rad51* and **(F)** Hif-1α target genes *AldoA, Bnip3*, and *Egln1* in hypoxia (2% O_2_) compared to normoxia (21% O_2_). β-actin (*Actb*) was used as housekeeping gene and normoxic samples as reference for mRNA relative expression quantification using the ΔΔCt method. ^*^p < 0.05, paired *t*-tests, *n* = 4.

Previous findings from our group (Sugrue et al., 2014) demonstrated that hypoxic MSCs display a Hif-1α-dependent increase in the protein levels of the DNA repair factors DNA-PKcs and Ligase IV (involved in NHEJ) while the levels of the HR factor Rad51 remained unchanged. Since the canonical function of Hif-1 is to control the expression of a myriad of hypoxia-responsive genes, we hypothesized that Hif-1α might be involved in mediating an increase in the mRNA expression level of DNA repair genes. Since our group has also previously shown that the immortalized mouse MSC cell lines MS5 and ST2 behave very similarly to primary mouse MSCs, in this study the MS5 cell line was chosen for a more detailed molecular investigation of the mechanism behind the effects of hypoxia on the DDR of MSCs. mRNA levels of *Hif1a, Prkdc, Lig4*, and *Rad51* were measured by real-time PCR in normoxic and hypoxic MSCs at different time-points after treatment with 10Gy of IR (Figure 1A). Surprisingly, hypoxia treatment did not result in an increase in mRNA levels of the genes analyzed, but instead it caused a mild but statistically significant decrease in the mRNA levels of *Rad51*. Significant up-regulation in the mRNA expression of all genes studied only occurred in response to IR, 24 h after irradiation in normoxic conditions. In hypoxia, a similar but less pronounced trend was observed, with only DNA-PKcs showing a statistically significant up-regulation. Despite the fact that hypoxia did not up-regulate the mRNA expression of *DNA-PKcs* and *DNA Ligase IV*, the previously described hypoxia-mediated increase in MS5 radio-resistance was reproduced (Figure 1B), indicating that this effect is not correlated with an increase of *DNA-PKcs* and *DNA Ligase IV* mRNA expression as had been previously hypothesized.

In order to perform a broader characterization of the effects of hypoxia on the transcription levels of DDR factors, customized commercial qPCR arrays were used in order to compare the mRNA expression of 87 genes belonging to the DDR signaling network in hypoxia (2% O_2_) relative to normoxia (21% O_2_). This array allows the analysis of genes involved in the ATM and ATR signaling cascade, different DNA repair pathways (such as nucleotide excision repair, base excision repair, mismatch repair and double strand break repair), as well as cell cycle control and apoptosis. In line with the previous results, hypoxia did not significantly affect the expression of the vast majority of these genes, with only one gene (*Mgmt*) up-regulated over 2-fold (although this difference did not show statistical significance) and only one gene (*Ogg1*) showing a significant but mild down-regulation (Figures 1C,D). Neither DNA-PKcs nor DNA Ligase IV (the proteins up-regulated as a result of the hypoxia treatment) showed significant differences at the level of mRNA, suggesting that the regulation of the NHEJ factors occurs at a post-translational level rather than at an mRNA expression level.

In order to confirm the qPCR array results, individual real-time PCRs for 53BP1 (*Tp53bp1*)*, Brca1*, DNA-PKcs (*Prkdc*), DNA Ligase IV (*Lig4*), *Mdc1*, and *Rad51* were performed, showing the same results (Figure 1E). *Rad51* was the only gene displaying a statistically significant (although mild) down-regulation in hypoxia compared to normoxia, similar to what had been observed in previous experiments (Figure 1A). In order to confirm the transcriptional responsiveness of the cells to hypoxia, mRNA expression of three well-known Hif-1α target genes, *AldoA, Bnip3*, and *Egln1* (Greijer et al., 2005) was analyzed. As expected, the hypoxia treatment resulted in all three genes showing a significant up-regulation (Figure 1F).

### Both subunits of the transcription factor Hif-1 are required for the hypoxia-induced increase in MS5 radio-resistance

**Figure 2:**
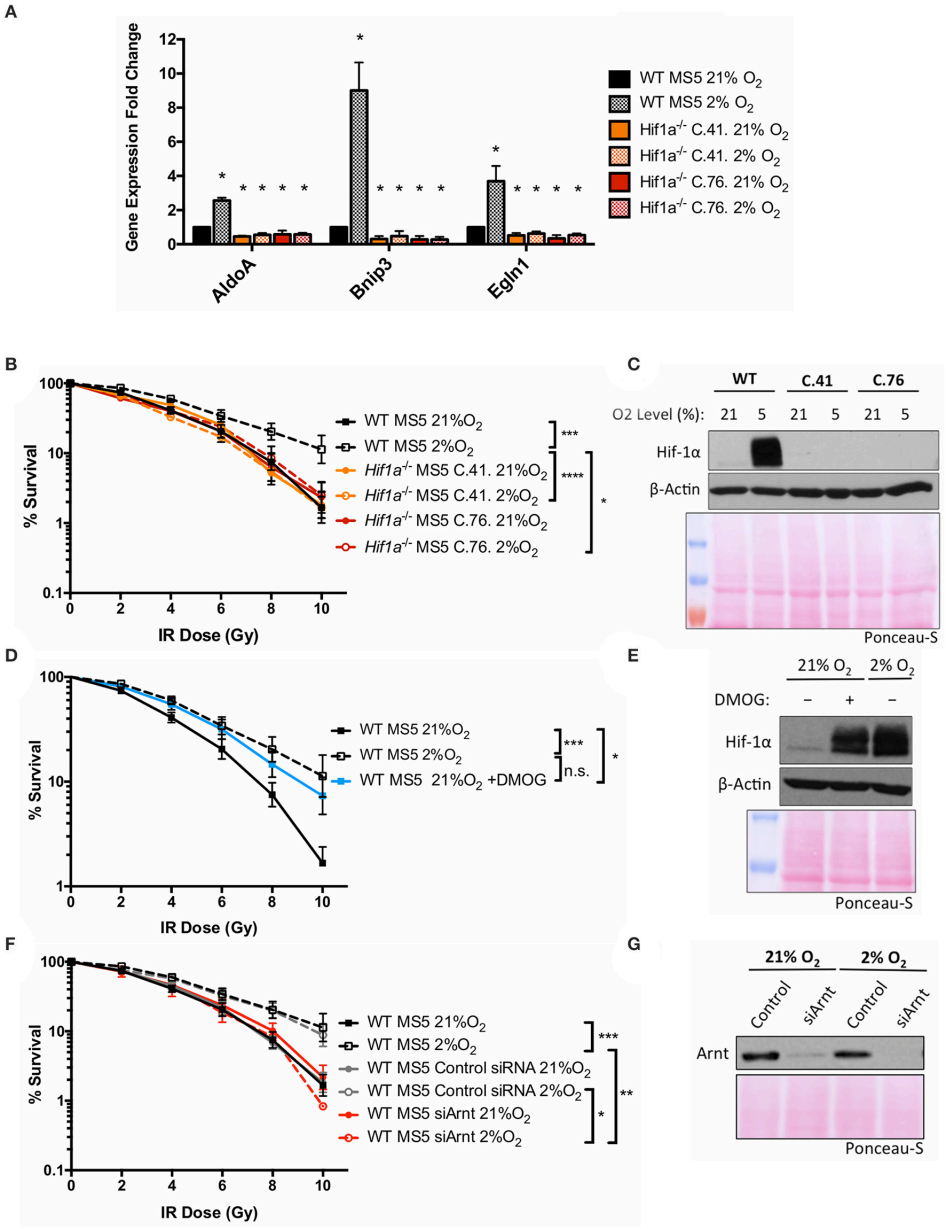
Both subunits of the transcription factor Hif-1 are required for the hypoxia-induced increase in MS5 radio-resistance. **(A)** Relative mRNA expression of the Hif-1α target genes *AldoA, Bnip3*, and *Egln3*, normalized against β*-Actin* and WT MS5 cultured in normoxia for mRNA relative expression quantification using the ΔΔCt method. ^*^p < 0.05, paired *t*-tests, *n* = 3. Clonogenic survival assay of **(B)** WT and *Hif1a*^−/−^ MS5 clones 41 and 76, **(D)** WT MS5 cells cultured in normoxia (21% O_2_), hypoxia (2% O_2_), or normoxia plus treatment with 500 μM DMOG for 48 h, and **(F)** untransfected, control and Arnt knock down WT MS5 cells. ^*^p < *0*.05, ^**^p < 0.01, ^***^p < 0.001, ^****^p < 0.0001, two-way ANOVA, *n* = 6. Representative Hif-1α western blot showing **(C)** WT MS5 cells and *Hif1a*^−/−^ MS5 clones 41 and 76 and **(E)** WT MS5 cells untreated or treated with 500 μM DMOG for 48 h. **(G)** Representative Arnt western blot of WT MS5 cells transfected with Control or siArnt siRNA.

To investigate the mechanism by which Hif-1α enhances the DDR of MS5 cells, a *Hif1a* knockout MS5 cell line was generated using CRISPR/Cas9 technology (detailed in Supplementary Materials). The newly generated *Hif1a*^−/−^ MS5 cell lines were used to study the effect of Hif-1α loss in MS5 radio-resistance. Two independent clones (41 and 76) were used in parallel in order to control for possible off-target effects resulting from the CRISPR/Cas9 *Hif1a* knockout protocol. First of all, disruption of the Hif-1 pathway by *Hif1a* deletion was confirmed by analyzing mRNA expression of known Hif-1α target genes (Figure 2A). *AldoA, Bnip3*, and *Egln1* are significantly up-regulated in hypoxic WT MS5 cells. However, *Hif1a* knockout not only completely abolished this up-regulation but also caused a significant decrease in the mRNA expression of these genes, irrespective of the oxygen levels. We then proceeded to analyze the effect of Hif-1α depletion on the radio-resistance of MS5 cells (Figures 2B,C). As previously shown, WT MS5 survival to increasing doses of ionizing radiation is enhanced in hypoxic conditions. In contrast, Hif-1α knockout prevented hypoxia-mediated increase in MS5 radio-resistance, indicating that this effect is Hif-1α-dependent (Figures 2B,C). In line with this result, treatment of normoxic WT MS5 cells with DMOG, a PHD inhibitor that prevents Hif-1α degradation in the presence of oxygen, resulted in a moderate increase of their radio-resistance in comparison with untreated cells, although not to the same level as the hypoxic untreated cells (Figures 2D,E). This might be explained by the fact that the increase in the levels of Hif-1α achieved through DMOG treatment was less than that found in hypoxic cells (Figure 2E) or that the regulation of HIFs in hypoxia is not only based on the prevention of their proteasomal degradation but also involves many regulatory post-translational modifications such as hydroxylation or phosphorylation, which cannot be mimicked by simply treating the cells with DMOG.

Since Hif-1α performs its functions through heterodimerization with Arnt, the requirement of this protein was also investigated. An Arnt-specific siRNA was used to deplete this protein from WT MS5 cells. This resulted in a reduction in the radio-resistance specifically in hypoxia (Figures 2F,G), becoming comparable to the survival levels displayed by the normoxic cells. This result suggests that not only Hif-1α, but also Arnt is required for the hypoxia-mediated increase in MS5 radio-resistance.

### Hif-1α ability to interact with both Arnt and DNA is required for its function in the DDR of mouse MS5 cells

Although the hypoxia-induced increase in MS5 radio-resistance is dependent on both Hif-1α and Arnt (the two subunits that compose the Hif-1 transcription factor), there is no effect of hypoxia on mRNA expression of a great number of the main genes that function in the DDR (Figures 1C,D). This is particularly surprising given that several of these factors (DNA-PKcs and DNA Ligase IV) show a Hif-1α-dependent increase of their protein levels in hypoxia (Sugrue et al., 2014). Despite the main canonical function of HIFs being to act as transcription factors, increasing numbers of studies indicate that Hif-1α is capable of modulating different pathways through direct interaction with other proteins to modulate their stability and/or function (Dang et al., 2011; Greer et al., 2012; Villa et al., 2014; Salminen et al., 2017). In light of this information, two hypotheses were formulated that could explain the previous results: either Hif-1α and Arnt have a different role in the DDR than their canonical transcription factor function (maybe controlling protein stability), or they are indeed acting as a transcription factor, but controlling the expression of unknown intermediary protein(s) that in turn affect the components of the DDR. In order to investigate these hypotheses, two different *Hif1a* mutant cDNA constructs were designed and stably expressed in *Hif1a*^−/−^ MS5 cells, and their ability to increase MS5 radio-resistance tested. The first mutant protein would lack the ability to interact with the DNA double helix (which would prevent it from performing its transcription factor function), while the second would not be able to interact with Arnt. Information regarding the design and generation of these cell lines can be found in the Supplementary Materials.

**Figure 3:**
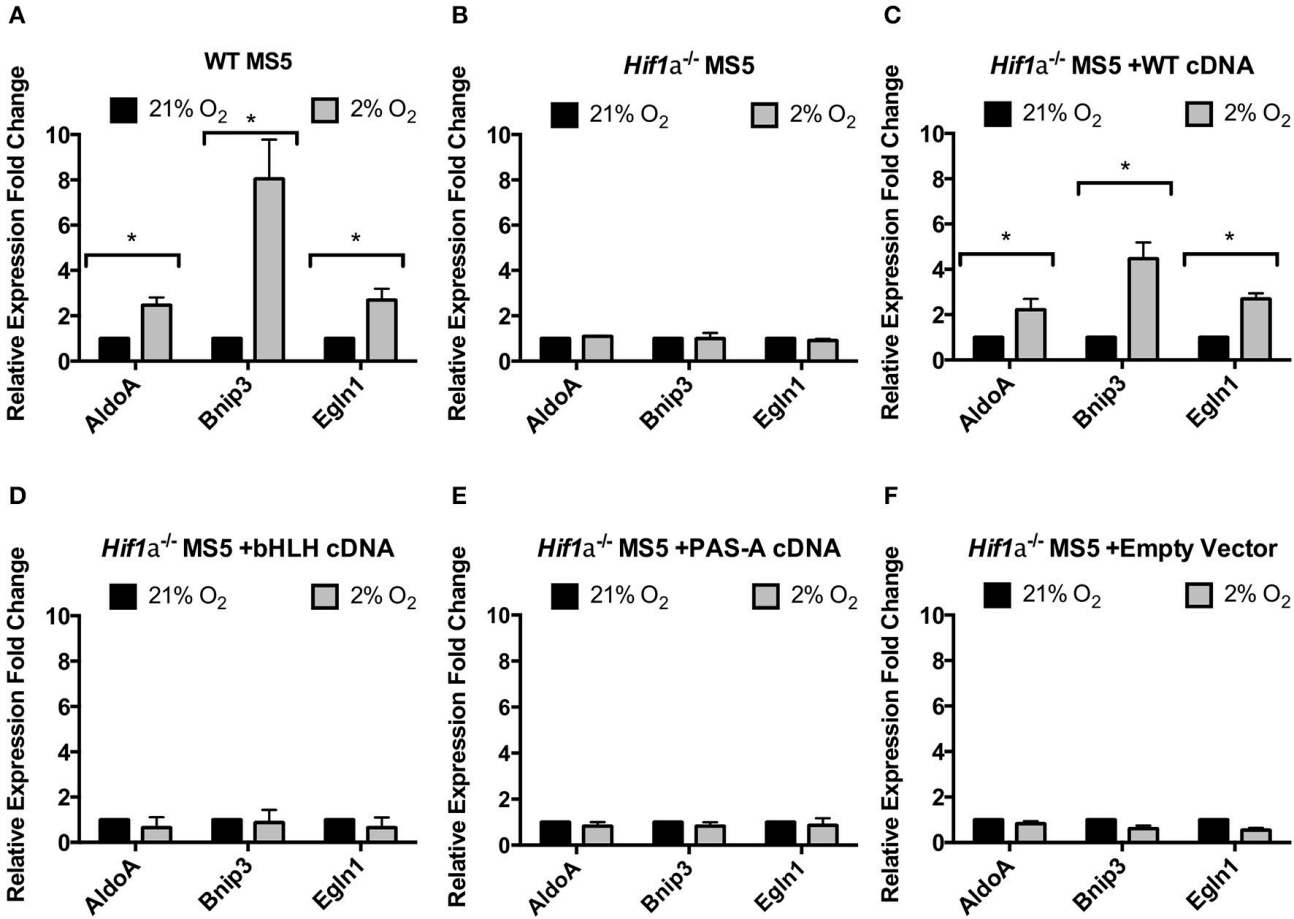
Functional properties of Hif1a cDNA mutants. Relative levels of Hif-1α target genes mRNA expression in **(A)** WT MS5 cell line, **(B)** *Hif1a*^−/−^ MS5 Clone 41 cell line, **(C)** *Hif1a*^−/−^ MS5 Clone 41 cell line transduced with a WT Hif-1a cDNA lentiviral construct, **(D)** *Hif1a*^−/−^ MS5 Clone 41 cell line transduced with a bHLH-mutated Hif1a cDNA lentiviral construct, and **(E)** *Hif1a*^−/−^ MS5 Clone 41 cell line transduced with a PAS-A-mutated Hif1a cDNA lentiviral construct, and **(F)** *Hif1a*^−/−^ MS5 Clone 41 cell line transduced with an empty lentiviral construct, cultured in 21 or 2% O_2_. Expression levels were calculated according to the ΔΔCt method normalizing against β-Actin (housekeeping gene) and considering 21% O_2_ as the reference condition for the calculations. ^*^p < 0.05 paired *t*-tests, *n* = 3.

Compared to the levels of the endogenous Hif-1α found in WT MS5 cells, all three versions of the exogenous Hif-1α protein (WT, bHLH-mutated, and PAS-A-mutated cDNA) were overexpressed (Supplementary Figure 4A), probably due to the hPGK promoter driving the expression in the lentiviral construct used. Encouragingly, despite this overexpression, in response to changes in the oxygen levels, the expression of the recombinant proteins was regulated similarly to the endogenous Hif-1α, and displayed a correct subcellular localization (Supplementary Figure 4B). Once the proper regulation of the recombinant Hif-1α proteins was confirmed, real-time PCR was used to assess their functionality. As previously shown, mRNA expression of the Hif-1α target genes *AldoA, Bnip3*, and *Egln1* is significantly up-regulated in hypoxic WT MS5 cells under hypoxic conditions, and this up-regulation is completely abolished when Hif-1α is knocked out (Figures 2A, 3A,B). Transduction of *Hif1a*^−/−^ MS5 cells with WT *Hif1a* cDNA rescued the mRNA expression of all three Hif-1α target genes (although this rescue was only partial in the case of *Bnip3*) (Figure 3C). In contrast, transduction with either bHLH-mutated or PAS-A-mutated *Hif1a* cDNA did not affect Hif-1α target gene mRNA expression (Figures 3D,E), indicating that their inability to interact with either DNA or Arnt rendered them unable to perform their transcription factor function. Cells transduced with an empty lentiviral vector were used as control to rule out any unspecific effects caused by the viral transduction and independent of the overexpressed cDNAs (Figure 3F).

**Figure 4:**
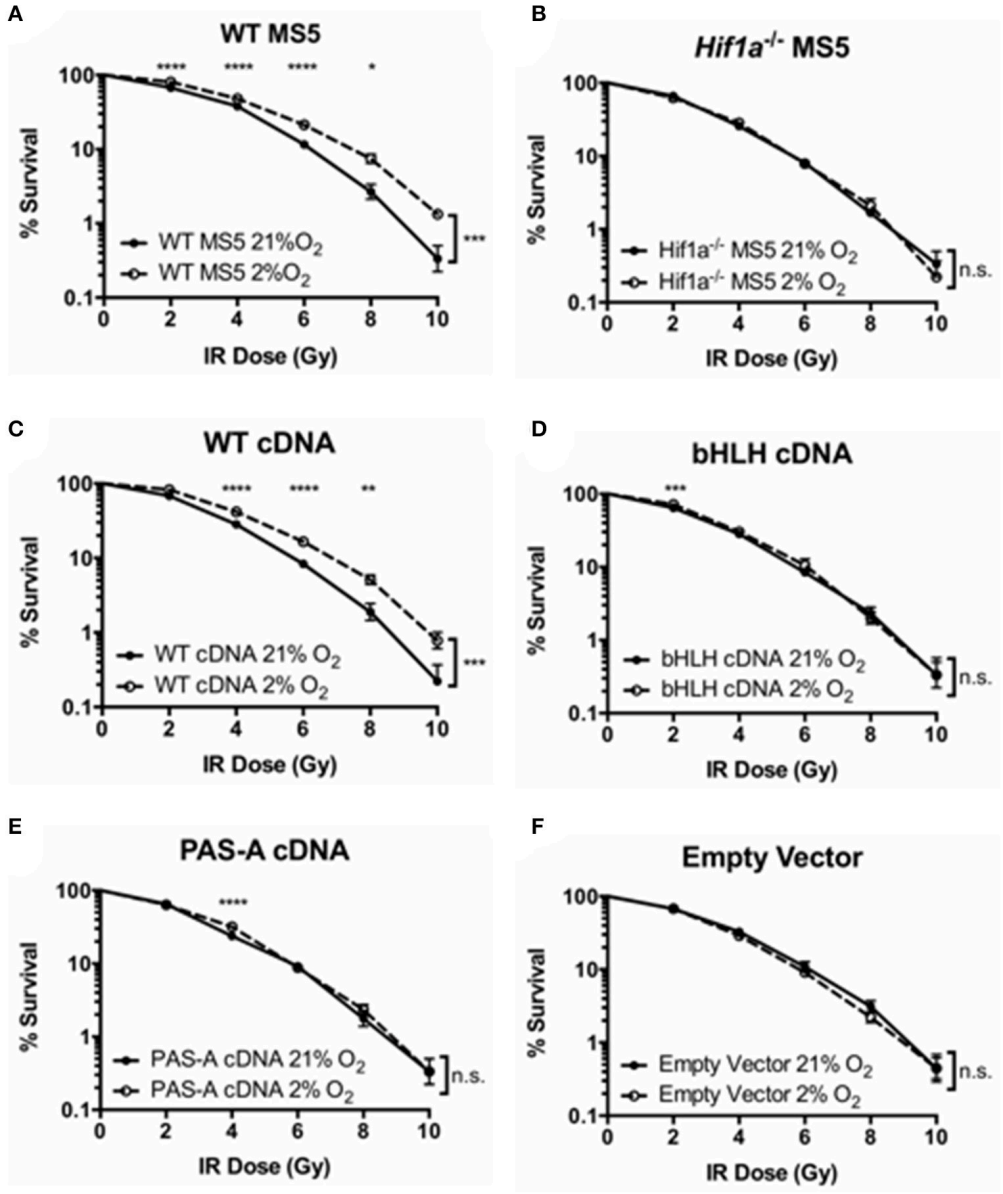
Effect of Hif-1α cDNA mutants on MS5 radio-resistance. Clonogenic survival assays of **(A)** WT MS5 cell line, **(B)** Hif-1α^−/−^ MS5 Clone 41 cell line, **(C)** *Hif1a*^−/−^ MS5 Clone 41 cell line transduced with a WT Hif-1α cDNA lentiviral construct, **(D)** *Hif1a*^−/−^ MS5 Clone 41 cell line transduced with a bHLH-mutated *Hif1a* cDNA lentiviral construct, and **(E)** *Hif1a*^−/−^ MS5 Clone 41 cell line transduced with a PAS-A-mutated *Hif1a* cDNA lentiviral construct, and **(F)** *Hif1a*^−/−^ MS5 Clone 41 cell line transduced with an empty lentiviral construct, cultured in 21 or 2% O_2_. ^*^p < 0.05, ^**^p < 0.01, ^***^p < 0.001, ^****^p < 0.0001 compared with normoxic samples, two-way ANOVA with Bonferroni post-test correction for the multiple comparisons, *n* = 9.

Next, the ability of these mutant proteins to function in the DDR of mouse MS5 cells (and that of the WT version to rescue the hypoxia-mediated increase in radio-resistance) was tested by clonogenic survival assays. First of all, WT and *Hif1a*^−/−^ MS5 cells, used as positive and negative controls, respectively, confirmed the previously shown increase in WT MS5 radio-resistance in hypoxia, and the loss of this effect caused by knocking out Hif-1α (Figures 4A,B). Interestingly, only the transduction of *Hif1a*^−/−^ MS5 cells with the WT *Hif1a* cDNA rescued the WT phenotype (Figure 4C), while none of the mutant proteins was able to mediate an increase in MS5 radio-resistance (Figures 4D,E), showing the same phenotype as the empty vector control (Figure 4F). These observations indicate that interaction of Hif-1α and Arnt to form the Hif-1 transcription factor, as well as its interaction with the DNA, are required for their function in the DDR of MS5 cells, which is most likely to involve regulation of gene expression by Hif-1.

**Figure 5:**
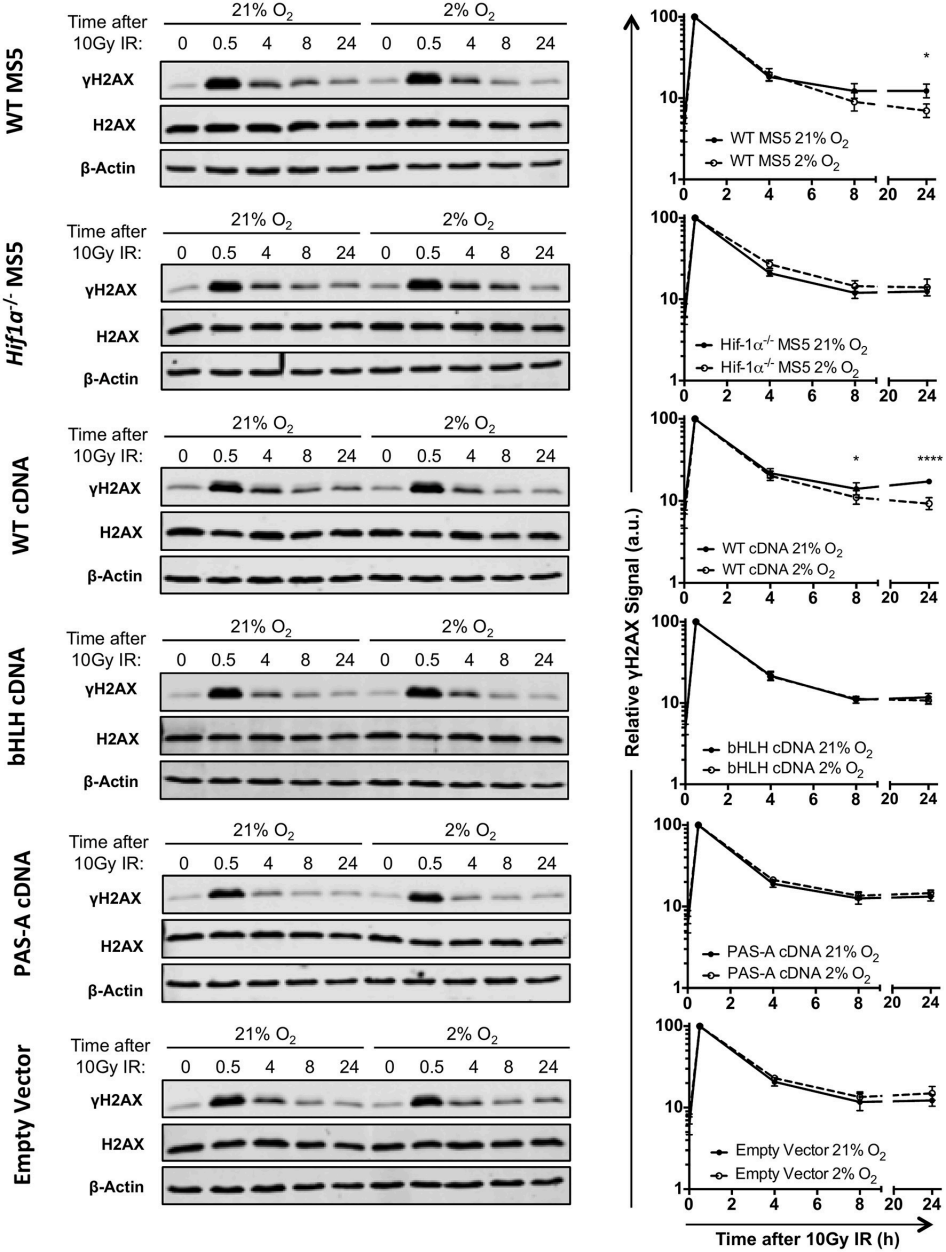
Effect of Hif-1α cDNA mutants on MS5 DSB repair kinetics. **(Left)** Representative western blots showing γH2AX, H2AX, and β-Actin levels in WT MS5 cell line, *Hif1a* ^−/−^ MS5 Clone 41 cell line, and *Hif1a*^−/−^ MS5 Clone 41 cell line transduced with and empty lentiviral vector, WT *Hif1a* cDNA bHLH-mutated Hif-1α cDNA and PAS-A-mutated *Hif1a* cDNA lentiviral constructs, cultured in 21 or 2% O_2_, 0–24 h after irradiation with 10Gy. **(Right)** Quantification of the relative γH2AX signal in the corresponding western blots, normalizing the signal against the 0.5 h timepoint (peak of the γH2AX signal) and using both H2AX and β-Actin as loading controls. ^*^p < 0.5, ^****^p < 0.0001, two-way ANOVA with Bonferroni post-test correction, *n* = 4.

In order to investigate whether Hif-1's ability to function as a transcription factor is also required for a more efficient DSB repair in hypoxia, and confirm the previous results obtained by clonogenic survival assay, DSB repair kinetics were analyzed by γH2AX western blot. Protein samples were harvested at different time points after treatment with 10Gy of IR, both in hypoxia (2% O_2_) and normoxia (21% O_2_). γH2AX signal (measured by infrared florescence of the bands obtained by western blotting) peaked 30 min after irradiation, and the signal progressively decreased over time indicating DSB repair (Figure 5). In hypoxic WT MS5 cells, the decrease in γH2AX signal occurred faster than in their normoxic counterparts, denoting a significantly faster repair of the DNA lesions. In contrast, *Hif1a*^−/−^ MS5 cells show similar DNA repair kinetics in both hypoxia and normoxia. The fact that γH2AX phosphorylation is not cleared faster in hypoxia in the absence of Hif-1α confirms that this protein is involved in enhancing the DNA repair efficiency of MS5 cells. In line with previous results, reconstitution using the WT *Hif1a* cDNA rescued the faster DSB repair observed in WT MS5 cells in hypoxia, while the two mutated cDNA versions showed the same phenotype as the *Hif1a*^−/−^ MS5 cells (Figure 5). Taken together, evidence obtained so far supports the hypothesis that, although Hif-1α may not be affecting the mRNA expression of the DDR factors studied so far, its ability to interact with Arnt to form the Hif-1 transcription factor and their interaction with DNA is critical for its role in enhancing the DDR of mouse MS5 cells.

### Comparison of WT and *Hif1a*^−/−^ MS5 proteome

**Figure 6:**
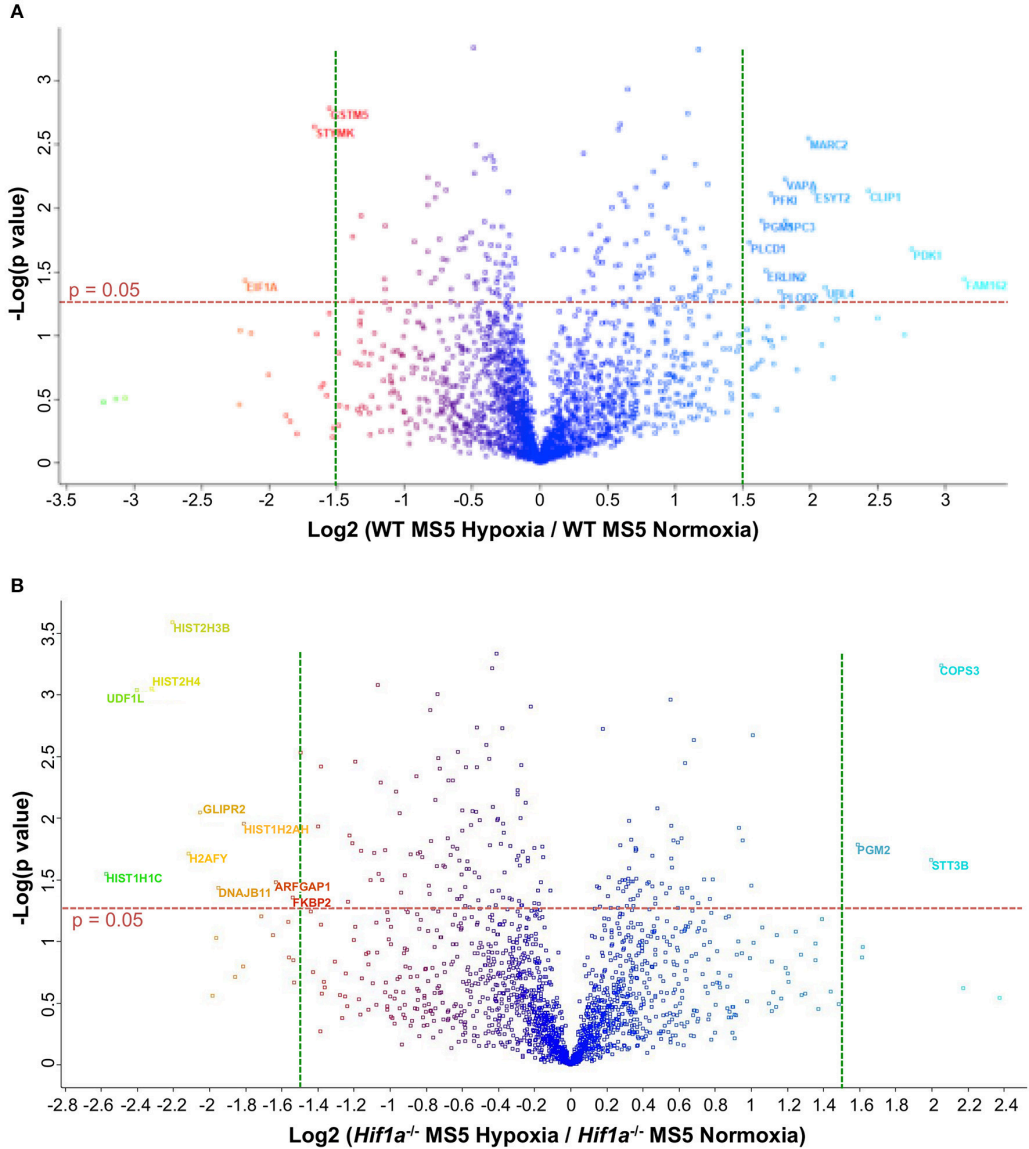
Volcano plots to compare effect of hypoxia on the proteome of WT and Hif1a^−/−^ MS5 cells. Gene expression fold changes of normoxic vs. hypoxic **(A)** WT MS5 and **(B)** *Hif1a*^−/−^ MS5 cells are plotted in the x axis vs. Log_10_ of the *p*-values derived from a *t*-test (y axis). Red horizontal dashed lines represents a *p*-value of 0.05 [–Log (0.05) = ~1.3], while green vertical lines indicate Log_2_ fold changes of −1.5 (left) and 1.5 (right). Names of proteins with *p* < 0.05 and Log_2_ fold changes >1.5 are indicated.

Hif-1α -mediated transcriptional regulation is highly complex and cell-type specific, therefore, the effect on the DDR might not be achieved through the regulation of one single intermediate factor but might be the result of a more intricate signaling network. For this reason, in order to investigate the global changes in protein abundance linked to Hif-1α activity in hypoxic MS5 cells, proteomic analyses were performed comparing WT MS5 cells and *Hif1a*^−/−^ cells in normoxia (21% O_2_) and hypoxia (2% O_2_). A total of ~2,200 different proteins could be identified in the WT samples, while only ~1,800 proteins were identified in the case of the *Hif1a*^−/−^ MS5 cells. Comparison of normoxic and hypoxic WT samples led to the detection of 173 proteins whose expression changes were statistically significant when a cut-off p value of 0.05 was applied (Supplementary Table 3). Of these, only 16 had Log_2_ (Fold Change) values higher than 1.5 (which corresponds to fold changes higher than ~3-fold), and are indicated in Figure 6A. In the case of *Hif1a*^−/−^ samples, 175 proteins were found to be significantly up- or down-regulated in normoxia compared to hypoxia (Supplementary Table 4), while only 14 had a Log_2_ (Fold Change) higher than 1.5 (Figure 6B). Despite the number of proteins significantly changed between the two pairs of samples, only 17 are common between the two, highlighting the importance of Hif-1α in mediating protein expression changes in response to hypoxia. Reproducibility amongst the sample replicates can be observed in the heat maps depicted in Supplementary Figure 5. In these, relative abundance (measured as ion current intensity) of proteins significantly changed using a cut-off *p*-value of 0.05 are shown for both the WT and the *Hif1a*^−/−^ MS5 groups of samples. Many well-known Hif-1α-regulated proteins, mainly (but not only) involved in metabolic pathways, such as AldoA, Anxa2, Eno1, Galk1, Gapdh, Hk2, Ldha, P4ha1, Pfkl, Pgk1, Pkm, Rhoa, or Tpi (Lee et al., 2004; Greijer et al., 2005; Hu et al., 2006) were found to be significantly up-regulated specifically in hypoxic WT MS5 cells but not in their *Hif1a*^−/−^ counterparts, therefore confirming the hypoxia-induced proteomic changes.

**Figure 7:**
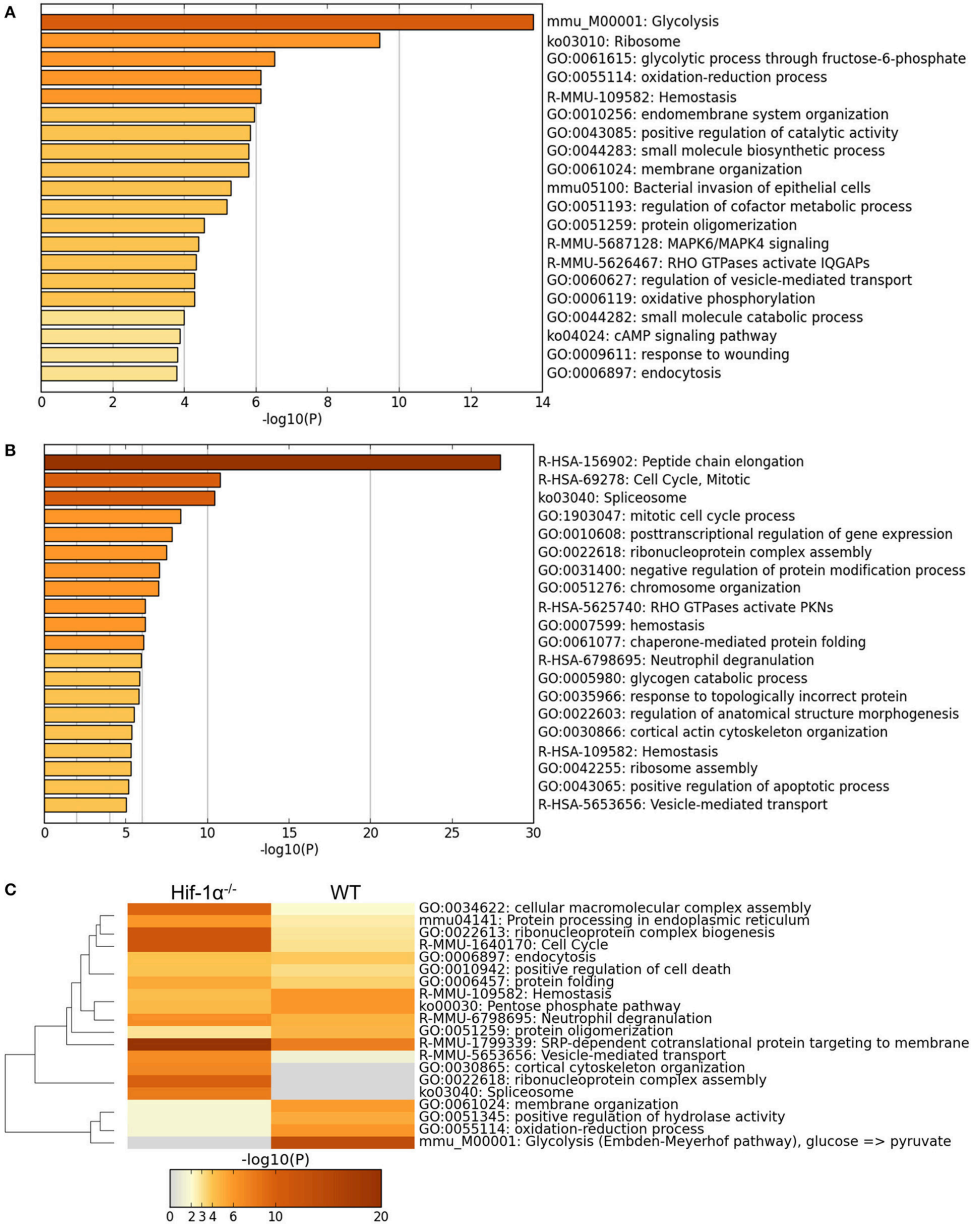
Main GO terms enriched in hypoxic WT and Hif1a^−/−^ MS5 samples. GO terms enriched in hypoxic WT MS5 samples when considering **(A)** the 173 proteins that change significantly according to the Log transformed intensity *t*-test between WT normoxic and hypoxic samples (*p* < 0.05), and **(B)** the 173 proteins that change significantly according to the Log transformed intensity *t*-test between *Hif1a*^−/−^ normoxic and hypoxic samples (*p* < 0.05). GO terms are shown in histogram plots in which the length of the bars corresponds to the statistical significance of the enrichment (–log *p*-value). **(C)** Heat map of GO terms enriched in hypoxic WT MS5 samples compared to hypoxic *Hif1a*^−/−^ MS5 samples when considering all proteins that change significantly according to the Log transformed intensity *t*-test with a *p*-value lower than 0.05. Graphs obtained using the *Metascape* online analysis tool.

Detailed analysis (GO term enrichment) of the proteins differentially regulated in WT cells in hypoxia (2% O_2_) compared to hypoxia (21% O_2_) identified many processes related with cellular adaptation to hypoxia such as glycolysis, oxidation-reduction processes or hemostasis, many of which were not found in the *Hif1a*^−/−^ samples (Figure 7), indicating as expected that Hif-1α plays an important role in the regulation of proteins belonging to these processes. Interestingly, GO term enrichment analysis with *Hif1a*^−/−^ MS5 samples showed enrichment of proteins belonging to the mitotic checkpoint and apoptosis pathways, which were mainly down-regulated and up-regulated, respectively, in a Hif-1α-independent fashion. However, the previously observed metabolic processes enriched in hypoxic WT MS5 cells (glycolysis, oxidation-reduction processes, etc.) were not found, indicating that the lack of Hif-1α prevents the metabolic switch that allows cells to adapt to low oxygen conditions (Figure 7). In addition, lack of Hif-1α-dependent regulation in Hif1a^−/−^ cells revealed changes in proteins involved in post-transcriptional and post-translational regulation, which are not present in WT cells and that might indicate a de-regulation of all these processes in the absence of Hif-1α.

A possible mechanism for the Hif-1-mediated increase in the protein levels of DDR factors without affecting their mRNA levels is through the transcriptional regulation of the expression of proteins involved in regulating protein stability and/or degradation, which would in turn mediate an increase in the half-life of these DDR proteins. For this reason, we focused on proteins involved in regulating protein stability and degradation. Interestingly, all protein ubiquitinases (Cul2, Uba5, and Ube2n) differentially regulated in WT MS5 samples showed down-regulation in hypoxia compared to normoxia, while the only deubiquitinase, Uchl5, was up-regulated (Supplementary Tables 3, 4). In addition, all members of the proteasome found in this list (Psma3, Psmc5, and Psmc6) were also expressed at lower levels in hypoxic WT MS5 cells. None of these proteins, however, was found to be differentially regulated in *Hif1a*^−/−^ MS5 cells, indicating that their regulation might be Hif-1α-dependent. In turn, unlike in WT MS5 cells, all proteins involved in the ubiquitin-related protein degradation pathway (Ube3a, Otub1, Nedd4) found to be expressed differently in normoxic and hypoxic *Hif1a*^−/−^ MS5 samples were up-regulated in hypoxia. Proteasome members showed a mixed response to hypoxia in these samples, although there was a tendency toward up-regulation of the majority of them (Psmd1, Psmb3, Psmd13).

## Discussion

MSCs are multipotent progenitors that reside in the hypoxic bone marrow niche (Eliasson and Jönsson, 2010; Mohyeldin et al., 2010), where hypoxia is thought to influence many aspects of their biology and functions (Jin et al., 2010; Tsai et al., 2012a,b; Liu et al., 2013; Prado-Lòpez et al., 2014). In many cancer cell types, hypoxia results in increased genetic instability and resistance to radio- and chemotherapy by impairing DNA repair mechanisms such as mismatch repair (MMR) and homologous recombination (HR) (Bindra et al., 2007; Bristow and Hill, 2008; Ruan et al., 2009; Scanlon and Glazer, 2015), and has been broadly related to poor prognosis. In contrast, we and others have shown that mouse MSCs cultured under hypoxic conditions display increased DNA repair efficiency as well as radio-resistance, and that this effect is dependent on Hif-1α (Sugrue et al., 2014). In this report, we have used the mouse MSC line MS5 to introduce genetic modifications allowing us to dissect the function of Hif-1α in the DDR. We have generated a stable HIF-1α KO MS5 MSC line in order to investigate the possible relationships between hypoxia and the DDR. Our results show that a complex picture emerges. Thus, although there is accumulation of proteins involved in the DDR, this does not appear to be regulated at the level of transcription of the corresponding genes.

Wu et al. recently described the crystal structure of the Hif-1α/Arnt heterodimer and its interaction with the DNA double helix (Wu et al., 2015). In this study, they were able to map the specific amino acids responsible for the interactions of Hif-1α with Arnt and the DNA double helix. By introducing the same mutations that we have introduced in the PAS-A domain (R170A and V191D), Wu et al. (2015) showed that the interaction between recombinant N-terminal portions of the Hif-1α and Arnt proteins was abolished *in vitro*. We have now been able to confirm this result *in vivo*, demonstrating that these two single amino acid substitutions disrupt Hif-1α function within the cells. In addition, we have also confirmed that the two amino acids identified by Wu et al. as the main interactors with the DNA double helix (K19 and R30) are essential for Hif-1α function and their substitution (K19Q and R30Q) also abolishes the ability of Hif-1α to regulate the expression of its target genes.

**Figure 8:**
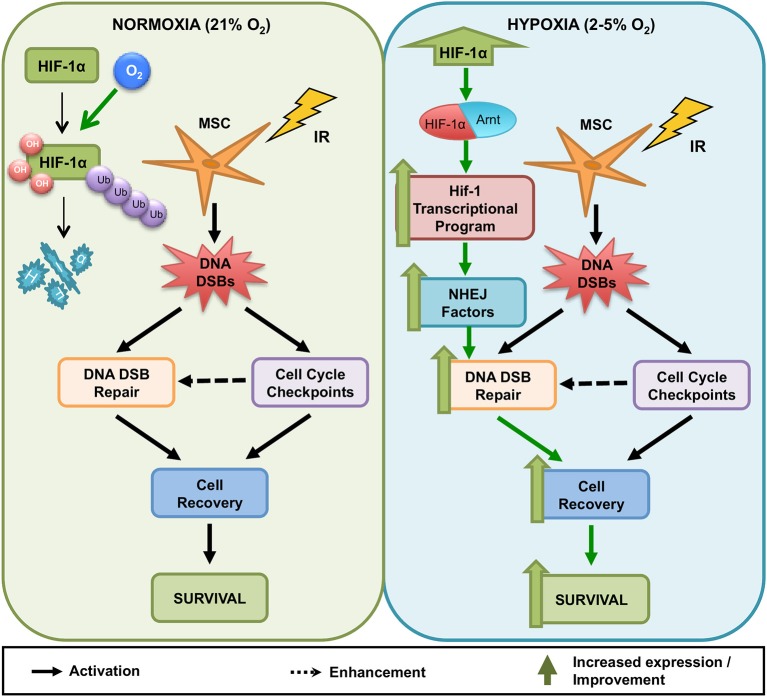
Model for the influence of hypoxia on the DNA Damage Response of mouse MSCs. In normoxia (21% O_2_, **Left)** and hypoxia (2–5% O_2_, **Right)**, irradiated MSCs activate DNA damage checkpoints and DNA DSB repair to resolve genotoxic DNA DSBs. Resolution of DNA DSBs likely enables irradiated MSCs to recover from cell cycle arrest and promotes MSC survival. Whereas in normoxia **(Left)**, high oxygen levels lead to the degradation of HIF-1α, in hypoxia **(Right)**, HIF-1α stabilization results in its accumulation in the cells and allows its interaction with Arnt to conform the Hif-1 transcription factor, which regulates the transcription of hypoxia-responsive genes. Activation of the Hif-1 transcriptional program in MSCs results in increased DNA DSB repair capacity, accelerating recovery from cell cycle arrest and improving long-term survival. This effect is dependent on the presence of a HIF-1α protein capable of binding to both DNA and HIF-1β.

Our data suggests that it is the Hif-1 transcription factor (and not only Hif-1α on its own) that enhances MS5 radio-resistance in hypoxia. Given the dependence of this effect on the integrity of the DNA binding domain (and therefore capacity to directly interact with the DNA), it is likely that Hif-1 is influencing the DDR of MS5 cells through transcriptional regulation. However, the fact that the vast majority of DDR factors analyzed are not transcriptionally regulated in response to hypoxia indicates that this is probably an indirect effect. Therefore, we propose a model (Figure 8) in which upon exposure to hypoxia, Hif-1α is stabilized and accumulates in the cells, being able to dimerize with Arnt to form the Hif-1 transcription factor, which will then drive the expression of unknown intermediary factor(s) that will in turn contribute to enhancing the DDR of MS5 cells and their survival post irradiation. We hypothesize that the increased protein levels of DNA-PKcs and DNA Ligase IV described previously by our group (Sugrue et al., 2014) might be due to a differential regulation of the protein turnover, and that perhaps Hif-1 coordinates the expression of proteins involved in controlling protein stability. Yet, attempts to measure DNA-PKcs and DNA Ligase IV protein half-life in normoxia and hypoxia and in the presence or absence of Hif-1α through cycloheximide treatment (an inhibitor of protein synthesis) were not successful (data not shown), and therefore this hypothesis could not be confirmed. However, preliminary proteomic analyses of the protein expression changes induced by hypoxia in both WT and *Hif1a*^−/−^ MS5 cells indicated that proteins involved in induction of protein degradation by the proteasome (such as ubiquitinases and members of the proteasome itself) were downregulated in hypoxic WT MS5 samples, while an opposite effect was observed in the case of *Hif1a*^−/−^ cells. Interestingly, some of these proteins have previously been shown to be involved in different aspects of the DDR (Zhao et al., 2007; Bencokova et al., 2009; Nakada et al., 2010; Cukras et al., 2014; Nishi et al., 2014; Mahanic et al., 2015; Xu et al., 2015). Although they do not constitute proof that this is the exact and only mechanism by which Hif-1 enhances the DDR of mouse MSCs in hypoxia, we believe these are interesting and encouraging observations worth noting. Further confirmation and investigation of these results will be necessary in order to establish the real impact of these changes on the Hif-1α-mediated enhancement of the DDR in hypoxic MSCs. Despite confirming the differential regulation of many well-known proteins controlled by Hif-1 (Lee et al., 2004; Greijer et al., 2005; Hu et al., 2006), we believe this preliminary proteomic analysis was not powerful enough to fully characterize the entire extent of proteomic changes induced by hypoxia in these cells, as demonstrated by the fact that known hypoxia-regulated proteins, such as Hif-1α itself, were not found to be significantly up-regulated in WT hypoxic samples, while western blot analysis of the same samples confirmed its differential regulation (data not shown). In future, more powerful proteomic analyses would be necessary in order to get the full picture of the proteomic changes induced by Hif-1 in MSCs.

Since the discovery of HIF-1 in 1992 by Semenza and Wang (1992), the mechanisms whereby HIF-1 regulates gene expression and the identity of all genes under its transcriptional control have been under continuous investigation. This has been done using different approaches ranging from genome-wide transcript profiling using expression micro-arrays to more recent chromatin immunoprecipitation coupled to high-throughput sequencing (ChIP-Seq) techniques (Hu et al., 2003; Greijer et al., 2005; Elvidge et al., 2006; Warnecke et al., 2008; Mole et al., 2009; Xia and Kung, 2009; Tanimoto et al., 2010; Schödel et al., 2011, 2012; Mimura et al., 2012; Tausendschön et al., 2015). One important lesson learnt from these studies is that HIF-1 dependent transcriptional control is highly complex and cell-type specific, being greatly influenced by chromatin status, epigenetic marks and direct or indirect interaction with other transcription factors (Schödel et al., 2013; Dengler et al., 2014). In line with this, hypoxia and HIFs have been shown to regulate and be regulated by many proteins involved in the DDR in many ways in different cell types (mainly cancer cells). To cite some examples, ATM activation in response to DNA damage has been shown to be reduced in Hif-1α knock-out murine fibroblasts (Wirthner et al., 2008), while loss of ATM prevented the stabilization and activity of HIF-1α under hypoxic conditions (Cam et al., 2010). Also, in human colon cancer cells, HIF-1α was shown to inhibit BRCA1 activity indirectly by counteracting C-MYC under hypoxic and normoxic conditions (Koshiji et al., 2004), while BRCA1 was found to enhance hypoxia-induced stabilization of HIF-1 (Kang et al., 2006). Similarly, Parp-1 directly interacts with the HIF-1α protein and contributes to its activation in several human cancer cell lines as well as murine embryonic fibroblasts (Rohwer et al., 2013). All these examples indicate that HIF-1α is notably interwoven with molecules centrally involved in the DDR (apart from many other cellular processes), which leads us to think that there is probably not a single mechanism for Hif-1-mediated enhancement of the DDR and the survival capacity of MSCs, but rather that this is most likely a complex process involving the regulation of a broad transcriptional program that will require further research in order to be fully understood.

The radio-resistance of MSCs can be therapeutically beneficial or unfavorable, depending on the clinical setting. Bone marrow transplantation (BMT) is a well-established therapy to treat patients with a variety of hematological cancers and immune disorders (Kim et al., 2013; Velardi et al., 2013). However, total body irradiation (TBI) and other preparative regimens required prior to BMT have important destructive effects on the supportive cell types that orchestrate recovery of the haematopoietic process (Williams and Gress, 2008; Cao et al., 2011). Indeed, reduction of the intensity of the preparative regimens has been shown to improve bone marrow HSC engraftment as well as the subsequent reconstitution of the immune system, probably due to the better preservation of the bone marrow and thymic microenvironments (Basara et al., 2002; Jiménez et al., 2005; Calvo-Asensio et al., 2017). Yet, while the protection of endogenous MSCs and/or the administration of exogenous MSCs are likely to have beneficial effects improving the outcomes of allogeneic BMT and in treating GVHD (Sugrue et al., 2017), MSC are also known to be recruited into the tumor microenvironment (Hanahan and Coussens, 2012; Kidd et al., 2012) where they are transformed into CAFs and promote tumorigenesis through various mechanisms (Kraman et al., 2010; Hanahan and Weinberg, 2011; Cuiffo and Karnoub, 2012; Hanahan and Coussens, 2012; Kidd et al., 2012; Barcellos-de-Souza et al., 2013). As shown herein, we confirm that Hif-1 is involved in MS5 radio-resistance in that its stable removal alters their response to DNA damage. Thus, Hif-1 could potentially be used as a therapeutic target to modulate MSC survival in response to IR. The growing interest in hypoxia and HIFs in the context of cancer therapy has resulted in the development of many drugs that allow either HIF-1's chemical inhibition or activation (Nagle and Zhou, 2006; Masoud and Li, 2015), which could be very useful for the development of these strategies. Further investigation into MSC radio-resistance and our understanding of how MSCs contribute to restore hematopoiesis, modulate the immune system and sustain cancer development in response to DNA damaging agents such as IR, will likely facilitate the development of more effective therapies for BMT and cancer.

## Conclusions

In conclusion, we have demonstrated that it is the transcription factor Hif-1 that enhances the radio resistance and DNA repair capacity of mouse MS5 cells. Our results indicate that Hif-1α acts in conjunction with Arnt to form the Hif-1 transcription factor, which requires its DNA-binding capacity to induce transcriptional changes in the cells that ultimately result in an increased radio-resistance and a faster DNA DSB repair. We hypothesize that it does so, at least partially, through an indirect mechanism involving the transcriptional regulation of factors capable of modulating the stability and/or function of DDR proteins. The investigation of the mechanisms behind the high radio-resistance of MSCs is essential for advancing our understanding of the role of MSCs in the reconstitution of the hematopoietic system following BMT and in the promotion of tumor growth and survival, which will allow the development of improved therapies.

## Author contributions

IC-A conception and design, collection and assembly of data, data analysis and interpretation, manuscript writing; ED collection and assembly of proteomic data; NL conception and design, data analysis and interpretation, manuscript writing, final approval of manuscript; RC conception and design, financial support, data analysis and interpretation, manuscript writing, final approval of manuscript.

### Conflict of interest statement

The authors declare that the research was conducted in the absence of any commercial or financial relationships that could be construed as a potential conflict of interest.
