# Supplementary figures and images for: The Transcription Factor Hif-1 Enhances the Radio-Resistance of Mouse MSCs
Source: Front Physiol. 2018 Apr 26;9:439. doi: 10.3389/fphys.2018.00439 (PMC5932323; doi:10.3389/fphys.2018.00439)

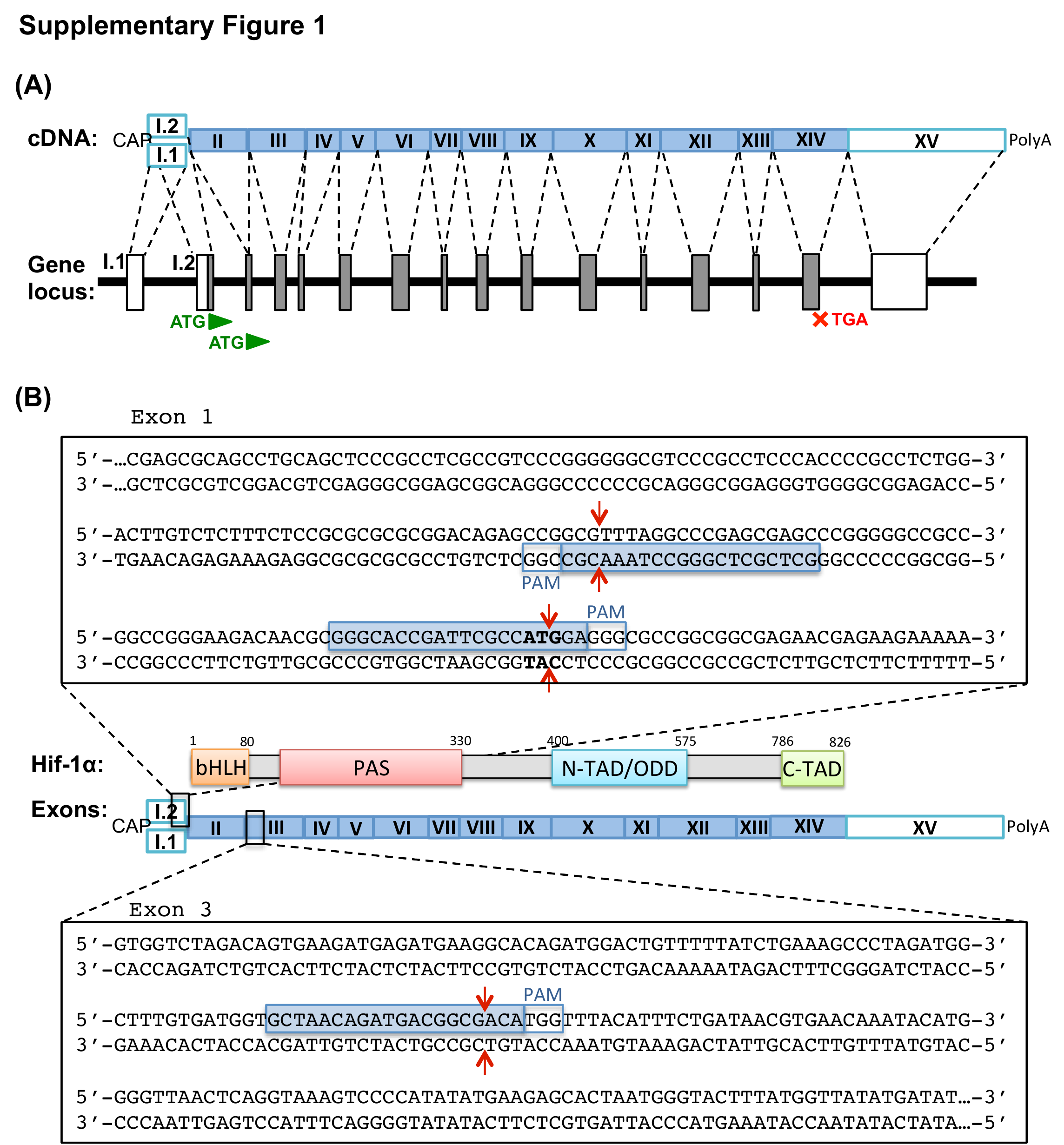

Supplement: Supplementary file 1 [file Image_1.TIFF]

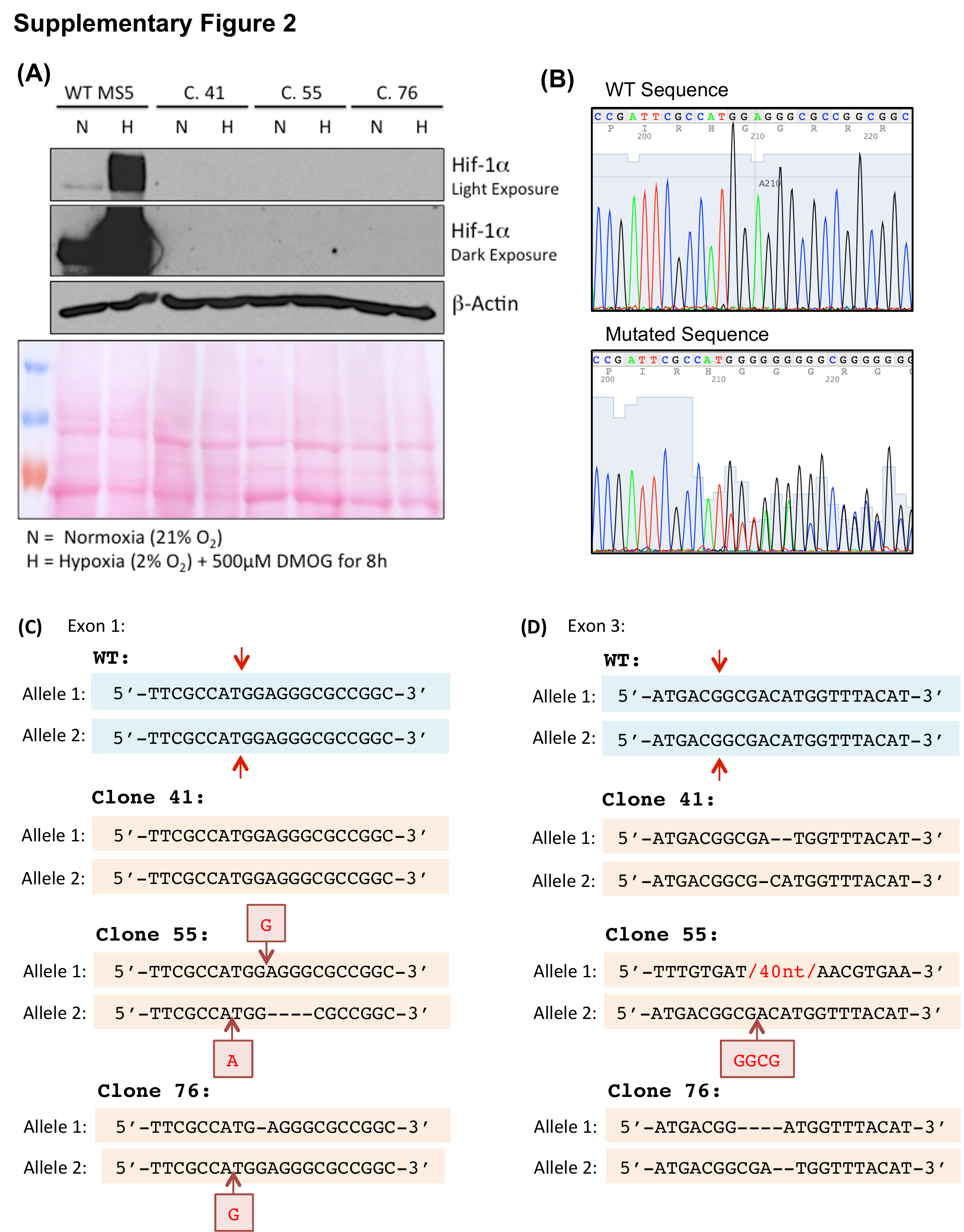

Supplement: Supplementary file 2 [file Image_2.TIFF]

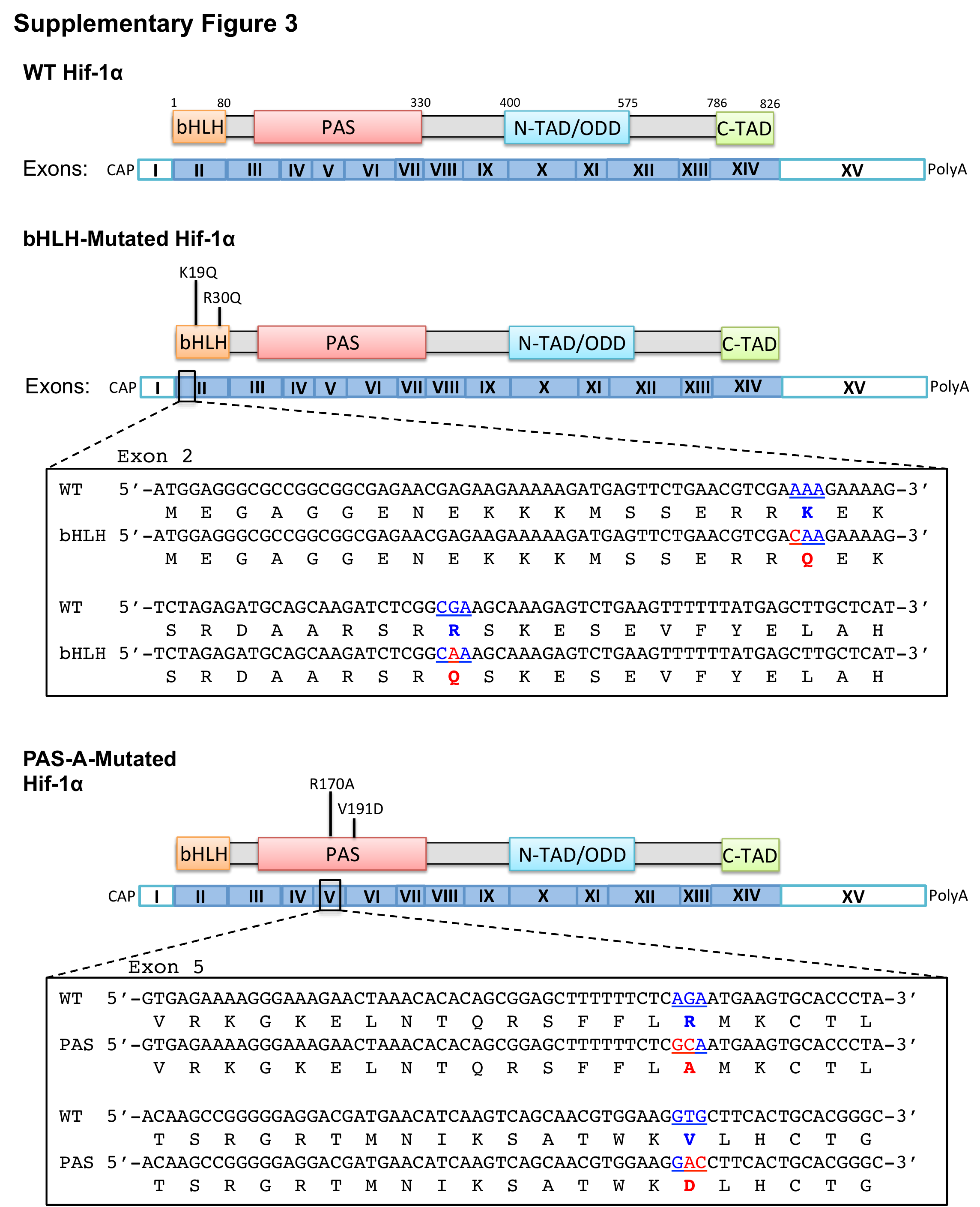

Supplement: Supplementary file 3 [file Image_3.TIFF]

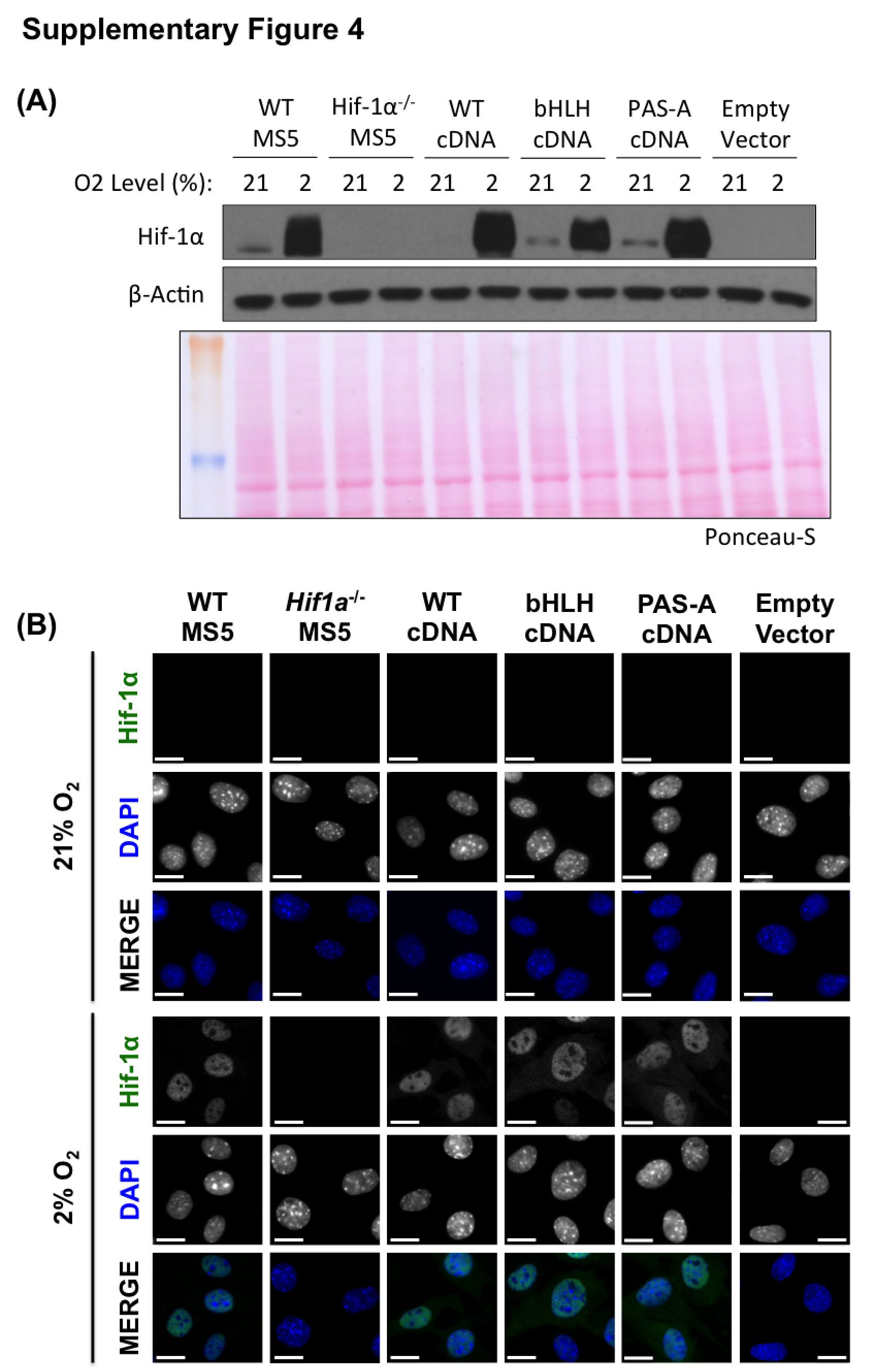

Supplement: Supplementary file 4 [file Image_4.TIF]

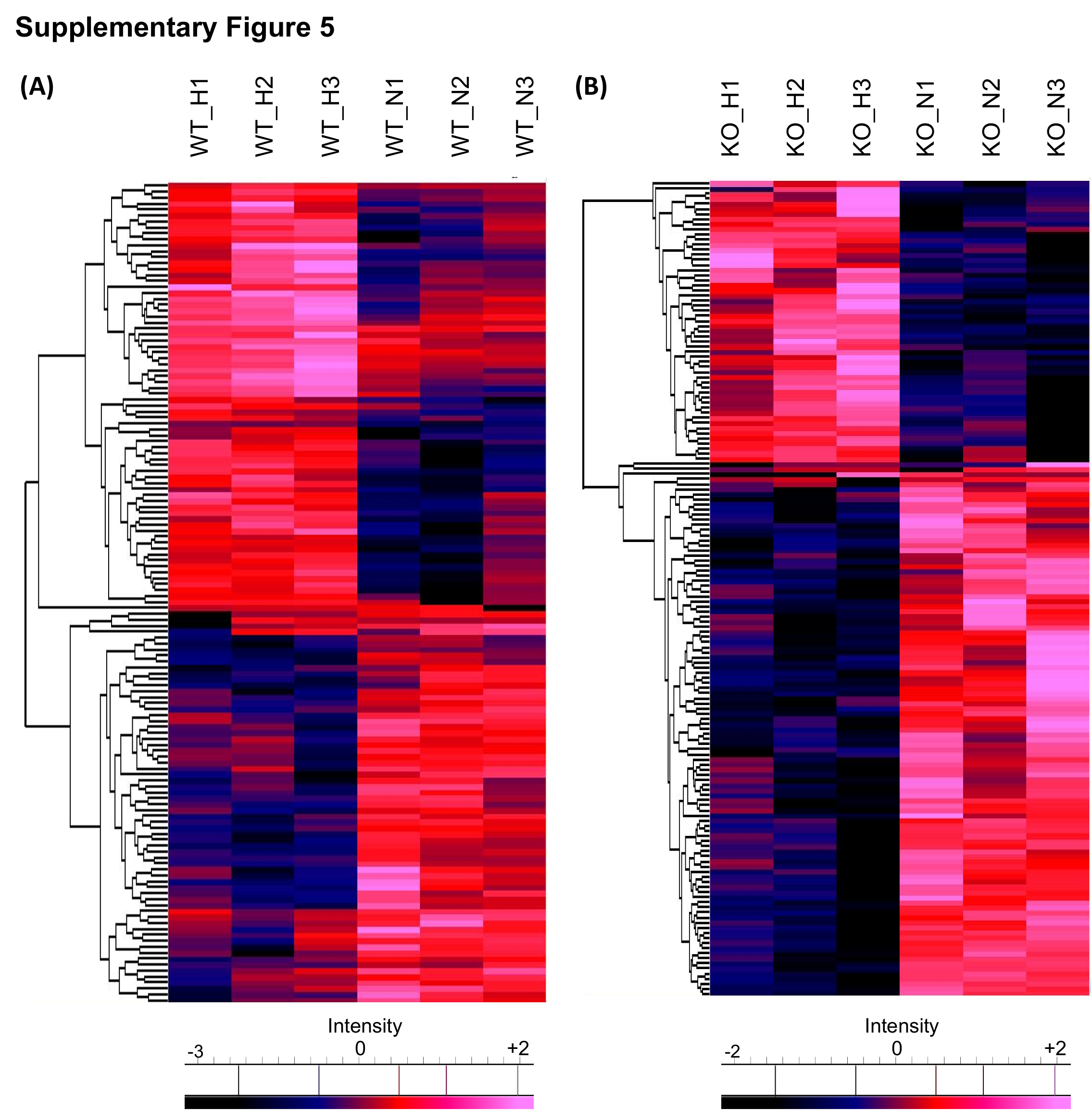

Supplement: Supplementary file 5 [file Image_5.TIFF]

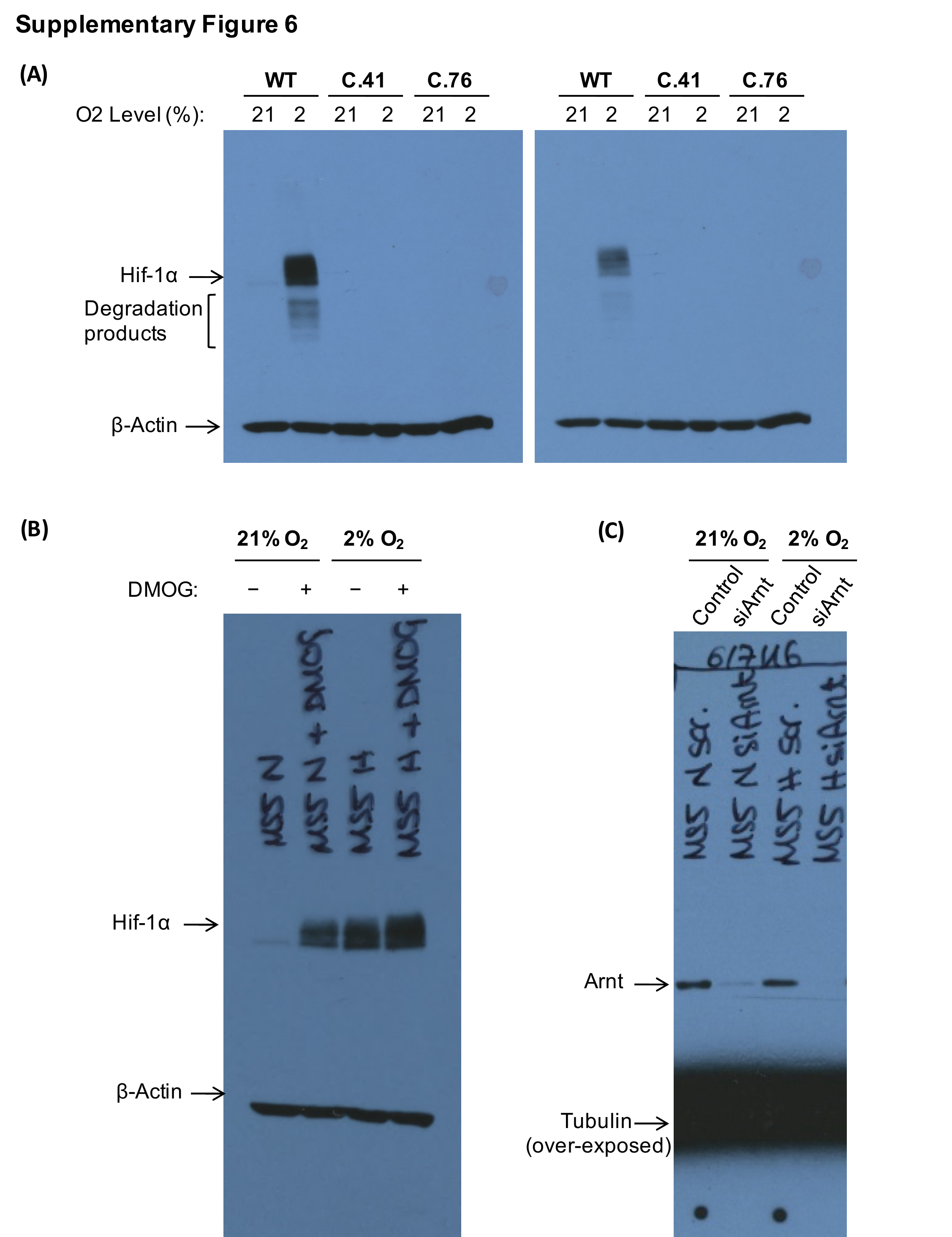

Supplement: Supplementary file 6 [file Image_6.TIFF]

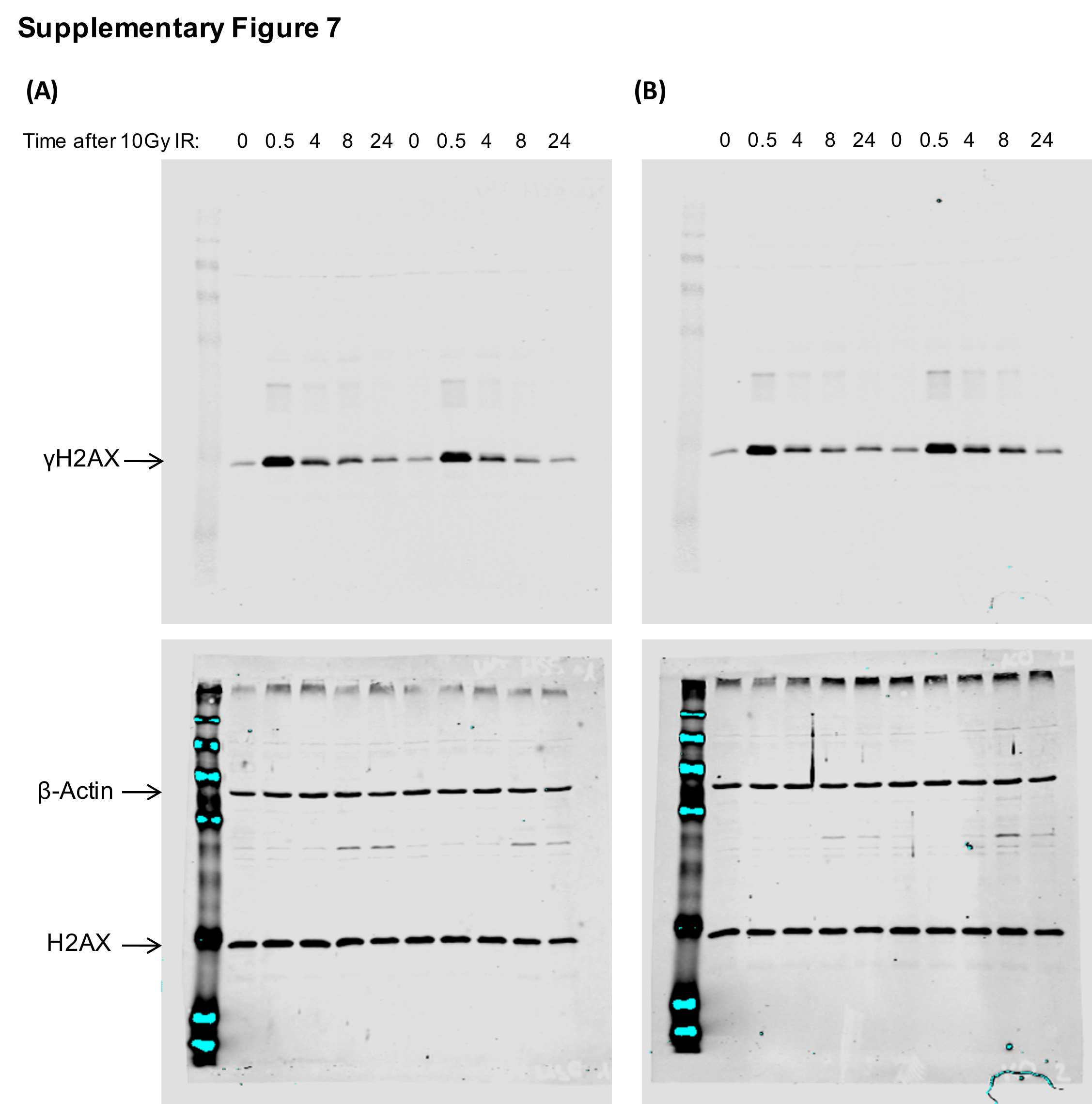

Supplement: Supplementary file 7 [file Image_7.TIFF]

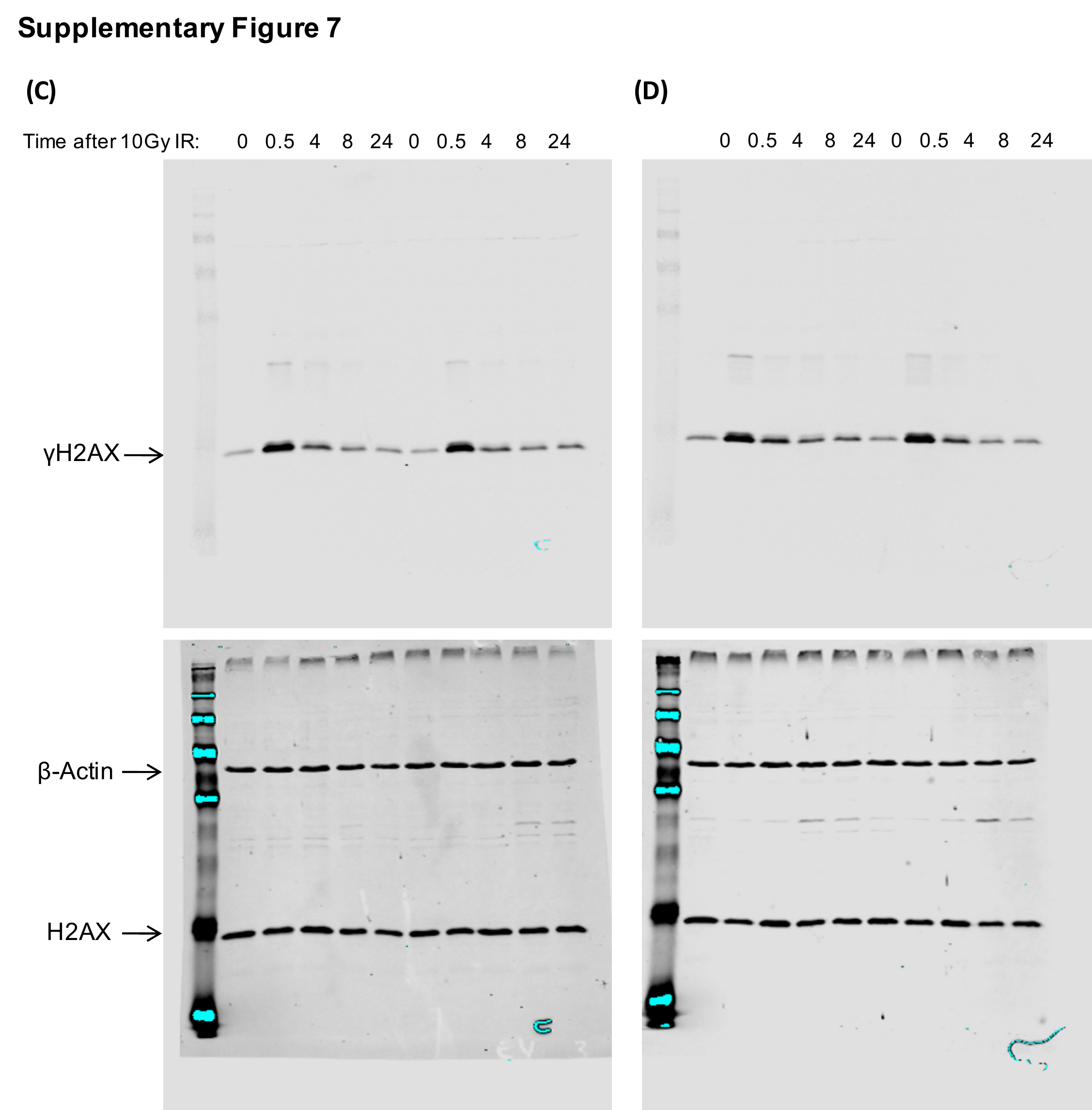

Supplement: Supplementary file 8 [file Image_8.TIFF]

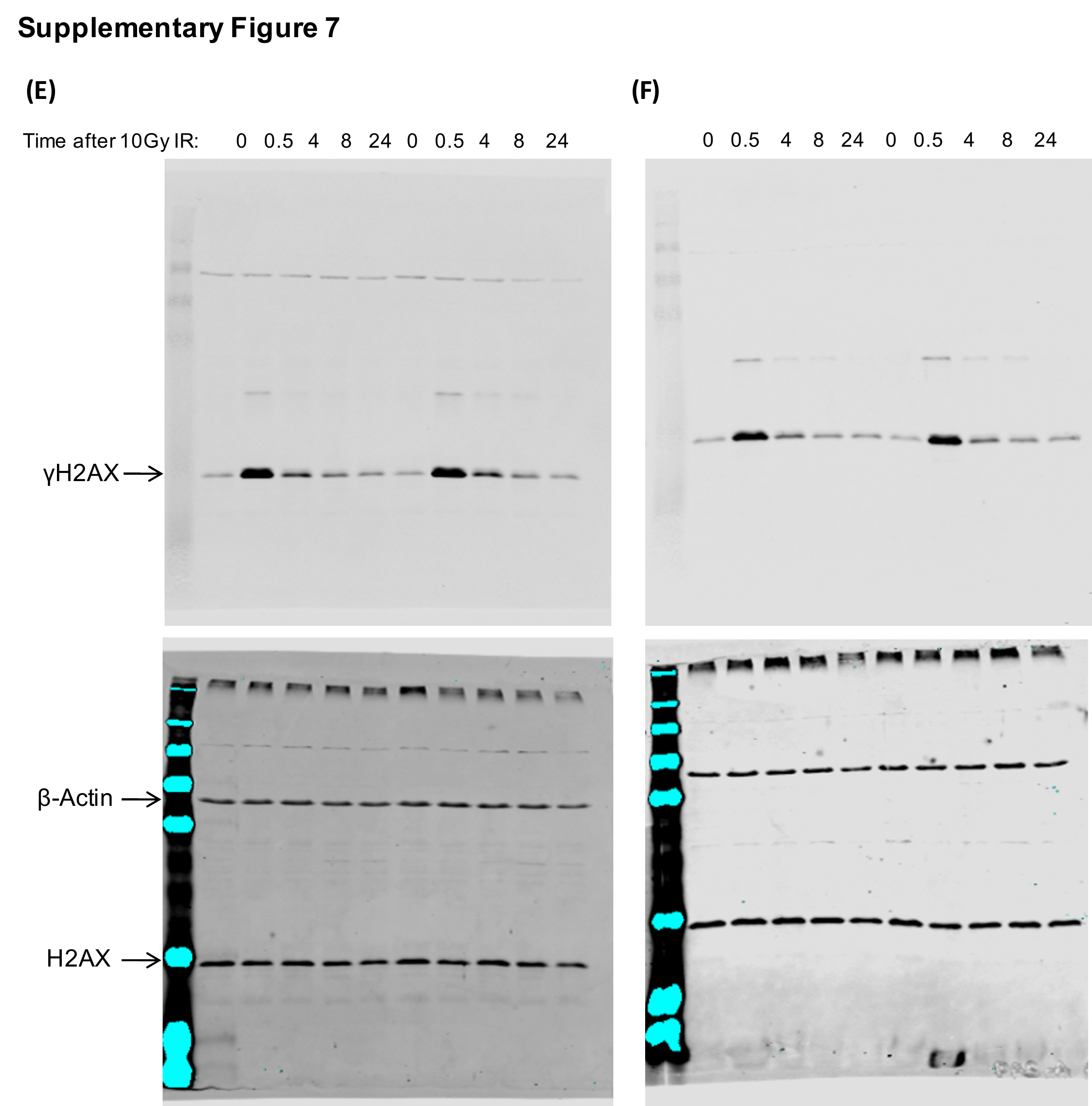

Supplement: Supplementary file 9 [file Image_9.TIFF]

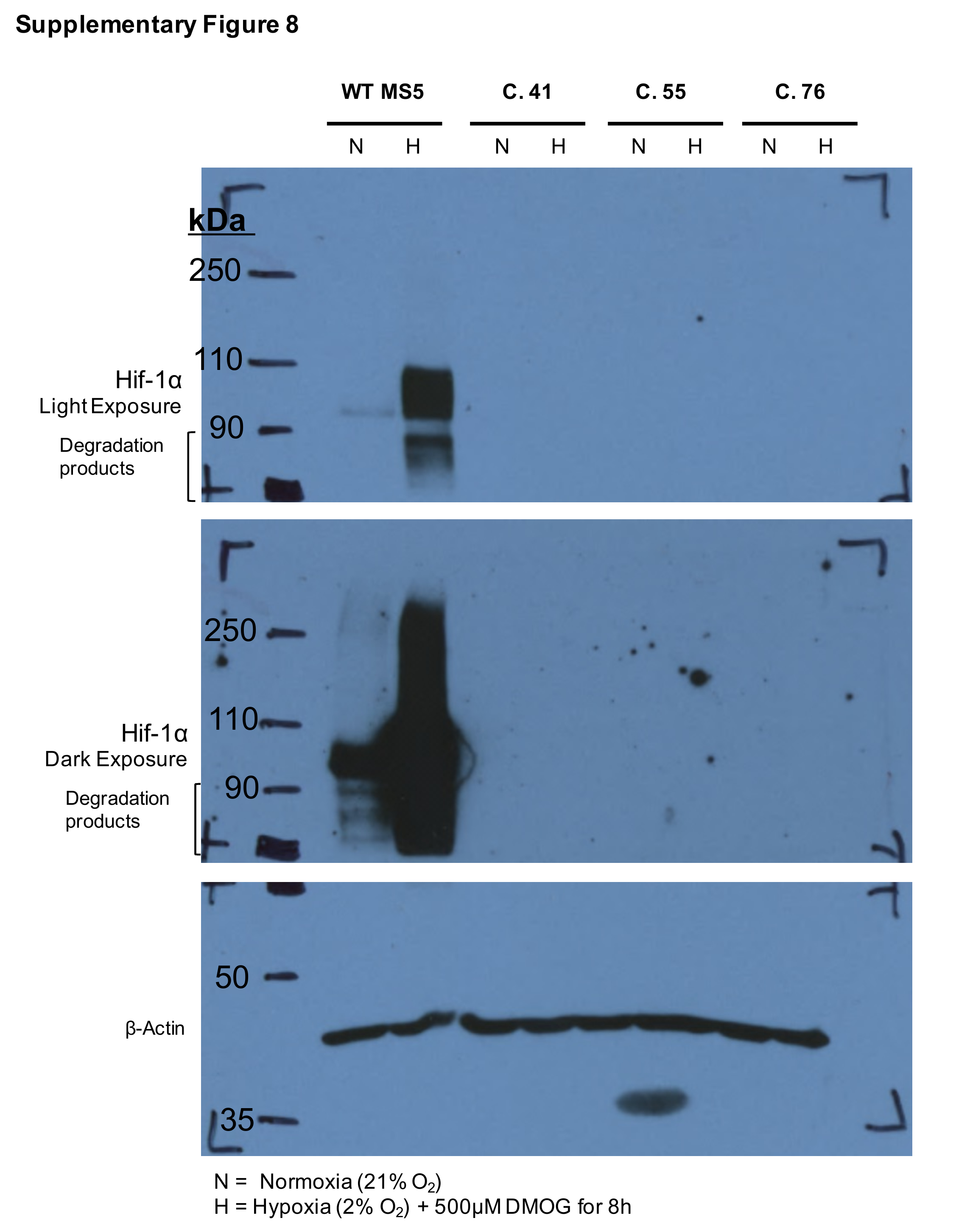

Supplement: Supplementary file 10 [file Image_10.TIFF]

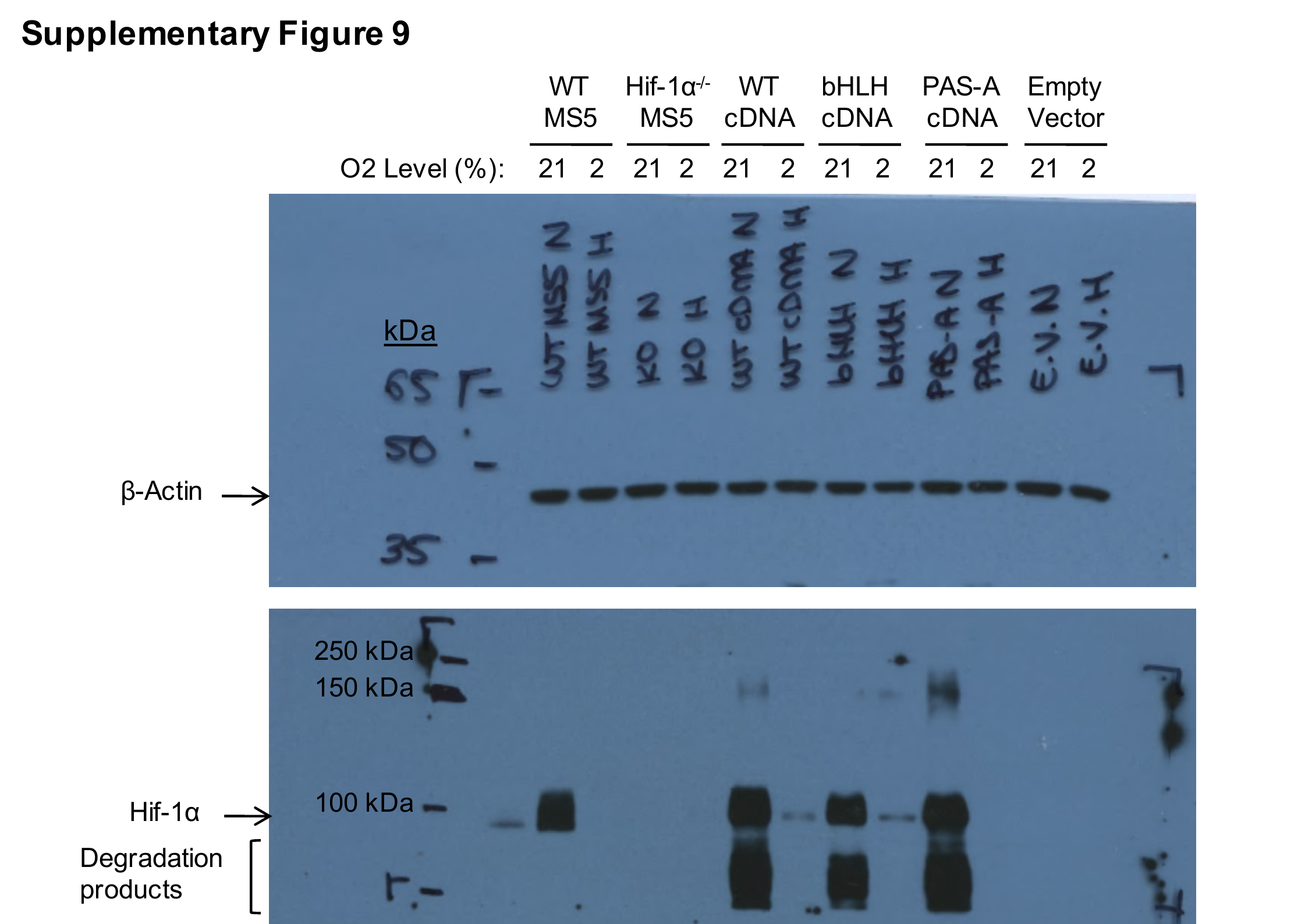

Supplement: Supplementary file 11 [file Image_11.TIFF]
