# Supplementary material for: The Transcription Factor Hif-1 Enhances the Radio-Resistance of Mouse MSCs
Source: Front Physiol. 2018 Apr 26;9:439. doi: 10.3389/fphys.2018.00439 (PMC5932323; doi:10.3389/fphys.2018.00439)
Supplement: Supplementary file 12 [file Data_Sheet_1.pdf]

Supplementary Material

The role if Hif-1 in enhancing the radio-resistance of mouse MSCs

Irene Calvo-Asensio[1,2], Eugène T. Dillon[3], Noel F. Lowndes[2*], Rhodri Ceredig[1*]

Correspondence:

Rhodri Ceredig
rhodri.ceredig@nuigalway.ie

Noel F. Lowndes
noel.lowndes@nuigalway.ie

Supplementary Data and Methods

1. Generation of a Hif-1α knockout MS5 cell line using CRISPR/Cas9 technology.

1.1. CRISPR/Cas9 Strategy

The *Hif1a* gene locus, which is located on mouse chromosome 12, contains 15 exons separated by 14 introns, dispersed over 45 kb (Semenza et al., 1996; Wenger and Gassmann, 1999). In 1997, Wenger et al. described two *Hif1a* mRNA isoforms in mice, resulting from two alternative first exons (exon 1.1 and exon 1.2) and differing mainly in their 5' UTR (Figure 3.2 A) (Wenger et al., 1997). When comparing the sequence information found in the NCBI database (https://www.ncbi.nlm.nih.gov), the exon 1.2-containing transcript (Transcript Variant 1) encodes a Hif-1α protein that is 12 amino acids longer in the N-terminal region than the exon 1.1-containing isoform (Transcript Variant 3). Transcript Variant 2, also described in the NCBI database, is nearly identical to Transcript Variant 1 except for the lack of three nucleotides in the N-terminal region and a single nucleotide substitution. According to this information, three different guide RNA sequences were designed, and the wild-type endonuclease version of the Cas9 enzyme was used in order to introduce double-strand breaks at the desired sites (Cong et al., 2013). As depicted in Supplementary Figure 1, two of the gRNAs were designed in exon 1.2, in order to target *Hif1a* transcript variants 1 and 2 at both sides of the translational start codon (ATG), in an attempt to generate a deletion that would impede transcription initiation at this site. Transcript variant 3 (whose expression is driven by a tissue-specific promoter (Wenger et al., 1998)) has its translational start codon in exon 2. However, this exon is relatively short (~190 bp) and no potential high quality gRNA sequences were found in it. For this reason, the third gRNA, which would target all mRNA isoforms, was designed in exon 3. Guide RNA specificity was tested by blasting the sequence against the mouse genome in order to avoid off-target effects, and its quality and predicted efficiency was assessed using two online available tools: MIT CRISPR

design tool (http://crispr.mit.edu/) and the DESKGEN genome-editing tool (https://www.deskgen.com).

1.2. CRISPR/Cas9 Methodology

1.2.1. Generation of CRISPR/Cas9 plasmids

Generation of *Hif1a*$^{-/-}$ MS5 cells was achieved using the pX330 WT Cas9 plasmid (obtained from Addgene, catalogue number #42230). Guide RNA sequences targeting the first and third exon of mouse *Hif1a* were designed manually as described above. Corresponding oligonucleotides (Supplementary Table 1) including overhangs for cloning were purchased from Sigma and reconstituted at a concentration of 100µM in nuclease-free H_2O. 25µl of each oligonucleotide were incubated at 95°C for 5 min and annealed by allowing them to cool down slowly until room temperature for approximately 2 hours. The oligonucleotides were then treated with T4 polynucleotide kinase (NEB) and re-annealed as previously described.

4ug of pX330 plasmid were linearized by digestion with BbsI restriction enzyme (Thermo Scientific) and treated with Calf Intestinal Alkaline Phosphatase (CIP, from NEB). The linearized plasmid was then purified using a PCR Clean Up kit from Thermo Scientific.

1µl of a 500nM solution of the annealed oligonucleotides was used for ligation into 50ng of the linearized pX330 vector using T4 Ligase (NEB). Ligation products were then transformed into competent Top10 *E. coli*, which were grown under Ampicillin selection. Clones corresponding to each guide RNA were picked and screened by BbsI digestion. Positive clones were sequenced to confirm the presence of the oligonucleotides and the absence of mutations using the pX330_seq primer (Supplementary Table 2).

Correct clones were purified from large-scale *E. coli* cultures using the NucleoBond® Xtra Midi / Maxi-prep kit from Macherey-Nagel and re-sequenced prior to transfection.

1.2.2. CRISPR plasmids transfection into MS5 cells

10^6 MS5 cells were seeded in a 10 cm petri dish with 10 ml of DMEM supplemented with 10% FBS and without Penicillin/Streptomycin. 24h later, ~75% confluent cells were transfected using Lipofectamine reagent (Thermo Fisher Scientific) with 3µg of each gRNA/Cas9 plasmid, 1µg of pLOX Puromycin resistance plasmid for selection of transfected cells and 0.5µg of eGFP expression plasmid to test for transfection efficiency. After incubation for 24h with the transfection mixture, cells were trypsinized and serial dilutions were performed in complete medium containing 10µM of Puromycin. Spare cells not used for the serial dilutions were used to assess transfection efficiency by flow cytometry. Cells were grown under selection for 48 hours before medium with puromycin was replaced with fresh medium and cells were cultured for 14 days until colonies were clearly visible.

1.2.3. Clone selection and screening

Single colonies were picked using cloning discs (Sigma) and transferred into 12 well plates. Cultures were scaled up until three wells of a 6-well plate were obtained per clone. One well was harvested for cryopreservation, the second one for protein extraction and screening and the third one for genomic DNA extraction and characterization of the mutations introduced.

Clones were screened for the presence or absence of the Hif-1α protein by western blotting, using treatment with 500µM of DMOG to prevent the degradation of the protein and ensure its detection. Positive clones missing the Hif-1α band were also further characterized by PCR and sequencing to determine the exact mutations introduced in each allele.

1.3. Generation and characterization of Hif-1α-/- MS5 cells. CRISPR/Cas9 results.

Mouse MS5 cells were transfected simultaneously with a pool of the three pX330 plasmids containing the three gRNA sequences designed, in order to increase targeting efficiency. Resulting clones were screened for the presence or absence of the Hif-1α protein by western blot. Hif-1α protein levels are very tightly regulated and its degradation is induced within seconds in the presence of oxygen. In order to avoid Hif-1α degradation during sample preparation that would result in false positives for the protein deletion, the 95 CRISPR clones selected were treated with 500µM DMOG, a cell permeable, competitive inhibitor of prolyl hydroxylase domain-containing proteins (such as the PHDs that hydroxylate HIF-1α in the presence of oxygen) prior to protein extraction for screening by western blot. Apart from this, genomic DNA from all these clones was also harvested in order to characterize the mutations introduced in their *Hif1a* loci. In order to characterize the targeting efficiency of the CRISPR/Cas9 strategy, 15 clones were randomly picked and gDNA regions to which the Cas9 was directed by the gRNAs were amplified by PCR and sequenced. Out of the 15 clones analyzed, 13 showed mutations in at least one exon, and only two were wild type, resulting in a targeting efficiency of ~87%. Most of these mutations had occurred at the exact sites where the Cas9 endonuclease was predicted to introduce double-strand breaks. Interestingly, when the forward primer complementary to exon 1 and the reverse primer complementary to exon 3 were used together, the gDNA from some clones gave rise to a ~400bp PCR product which was absent in the WT MS5 gDNA used as control. Sequencing of this DNA fragment revealed that it was the result of a ~20kb deletion between the Cas9 cut sites in exons 1.2 and 3 (data not shown). However, no mutations were detected at the site where the first gRNA would direct the Cas9 to cut, indicating that this particular gRNA had not worked properly. In order to investigate the reasons for this, the MS5 *Hif1a* cDNA was isolated and most of it (except for the 3'UTR and the beginning of the 5'UTR) was sequenced and compared to the reference sequence used to design the gRNAs. A total of 10 single nucleotide changes, a 1 nucleotide deletion (in the 5'UTR, so not affecting reading frame) and a 42 nucleotide deletion were detected, which result in 4 amino acid substitutions and the deletion of a fragment of 14 amino acids in the protein sequence. This 14 amino acid deletion falls in the ODD domain and had been previously described by Wenger et al. in 1996 (Wenger et al., 1996).

Unfortunately, two of the single nucleotide changes were found in the sequence of the first gRNA, explaining why it did not work.

Supplementary Figure 2 shows an example of three of the *Hif1a* knockout clones obtained: clones 41, 55 and 76. Sequencing of their gDNA (Supplementary Figure 2 B) allowed characterizing the specific mutations present in each of their two alleles (Supplementary Figure 2 C, D). Exon 3 of clone 41 presented a single-nucleotide and a two-nucleotide deletion in each allele, respectively, resulting in the disruption of the reading frame of the protein and, eventually, the introduction of a premature stop codon. Similarly, clone 55 showed two single-nucleotide insertions in exon 1 and a 40-nucleotide deletion and a 4-nucleotide insertion in each allele in exon 3. Finally, clone 76 presented a single-nucleotide deletion and a single-nucleotide insertion in exon 1 and a two-nucleotide and a four-nucleotide deletions in the two alleles in exon 3. Two of these *Hif1a*$^{-/-}$ clones (41 and 76) were selected as two independent clones for subsequent experiments.

2. Generation of Hif-1α mutant MS5 cell lines.

2.1. Design of Hif1a mutant constructs.

In December 2015, Wu et al. published a partial crystal structure of the Hif-1 transcription factor interacting with the DNA double helix (Wu et al., 2015). This crystal structure is composed of the N-terminal segments of both Hif-1α and Arnt, thereby allowing the mapping of the main specific residues that mediate both the interaction between Hif-1α and Arnt an also their interaction with DNA. Both Hif-1α and Arnt are members of the helix–loop–helix-PER-ARNT-SIM (bHLH-PAS) family, and as such they are composed of an amino-terminal bHLH domain for DNA binding, PAS domains (PAS-A and PAS-B) that allow dimerization, and transactivation domains (TADs) in order to modulate their function. In addition to these, Hif-1α contains an Oxygen-Dependent Degradation Domain (ODDD) which is critical for its regulation and function (Supplementary Figure 3 A) (Kewley et al., 2004). In order to prevent Hif-1α interaction with DNA, two point mutations were introduced in its bHLH domain that would result the following two amino acid substitutions: K19Q and R30Q (Supplementary Figure 3 B). These are the two positively charged amino acids that directly interact with the negatively charged DNA molecule. According to the COSMIC Database, these two specific substitutions have been found in cancer patients in which Hif-1α function is altered (Forbes et al., 2015; Wu et al., 2015). Wu et al. 2015 also demonstrated that interaction between Hif-1α and Arnt could be abolished by mutating two single residues found in the PAS-A domain of Hif-1α: R170A and V191D (Wu et al., 2015), resulting from two double-nucleotide mutations in the Hif-1α cDNA (Supplementary Figure 3 C).

2.2. ***Methods for generation and transduction of lentiviral vectors***

2.2.1. *Hif1a cDNA cloning into pLENTI PGK Blast plasmid*

Hif-1α cDNA was isolated from whole MS5 cDNA by PCR, using specific primers complementary to the start of the coding sequence in Exon 1.2 (F primer) and to the end of the coding sequence in Exon 15 (R primer) (Supplementary Table 2), finishing in the last nucleotide before the transcriptional stop codon. In addition, these primers contained overhangs corresponding to attB sites to allow cloning by Gateway cloning through a BP reaction. Amplified *Hif1a* cDNA was run on an agarose gel in order to assess specificity of the reaction. The band corresponding to the *Hif1a* cDNA predicted size (~2.5kbp) was extracted from the gel, sequenced and cloned into pDONR201 plasmid (Invitrogen) by gateway cloning.

Different clones were screened by digestion and sequencing to confirm the absence of mutations caused during the PCR amplification or the cloning process. Correct clones were purified from large-scale *E. coli* cultures using the NucleoBond® Xtra Midi / Maxi-prep kit from Macherey-Nagel and re-sequenced.

bHLH- and PAS-A-mutated *Hif1a* cDNA versions were generated by site-directed mutagenesis as detailed below. Resulting plasmids were screened by sequencing and correct clones were again purified from large-scale *E. coli* cultures as previously described, and re-sequenced.

WT and mutated versions of the *Hif1a* cDNA contained in pDONR201 plasmids were cloned into the pLENTI PGK Blast plasmid (Adgene), which is a lentiviral mammalian expression vector driven by the hPGK promoter and containing a Blasticidin resistance cassette. Gateway cloning was performed through an LR reaction using the LR recombinase enzyme obtained from Invitrogen. The clones obtained were screened by enzymatic restriction and sequencing, and correct clones were purified from large-scale E. coli cultures using the NucleoBond® Xtra Maxi-prep kit (Macherey-Nagel) to achieve higher plasmid yield and concentration and sequenced.

2.2.2. *Site-directed mutagenesis*

In order to introduce specific mutations in the Hif-1α cDNA, complementary pairs of primers containing the desired nucleotide changes were designed (Supplementary Table 2). The mutagenesis reaction was carried out using KOD polymerase according to the manufacturer's guidelines, using an Eppendorf Mastercycler ep Gradient S thermocycler. The products obtained were checked on a 1% Agarose gel before addition of 1.5μl of DpnI restriction enzyme directly to the mutagenesis reactions in order to eliminate the original plasmid template molecules that (unlike the newly generated mutant plasmids) are methylated and thus prone to digestion with the enzyme. Digestion reactions were incubated for 12-16h at 37°C and then transformed into competent Top10 *E. coli*. Single colonies were picked and plasmids were checked for the presence of the desired mutations by sequencing.

2.2.3. Lentiviral particle generation

$5x10^5$ HEK293T cells were seeded per 10cm petri dish and cultured until they reached a ~70% confluency level. Each of the pLENTI PGK Blast vectors containing the different versions of the Hif-1α cDNA was mixed with the three packaging plasmids psPAX2, pMS2.g and Rev plasmids as indicated in Table 3.6, and diluted in a 150mM NaCl solution up to a 250µl volume. 40µl JET-PEI transfection reagent (Polyplus) were mixed with 210µl of a 150mM NaCl solution and incubated at room temperature for 5min prior to mixing it with the plasmid mix previously prepared and incubating this new mixture for 20min at room temperature.

HEK293T cell growth medium was replaced with 10ml fresh DMEM containing 10% heat-inactivated FBS and 1% penicillin-streptomycin solution. After incubation, pDNA/JET-PEI mix was added gently to the tissue culture plates, and mixed well. Cells were then cultured for 24-48 hours at 37^oC and 5% CO_2 in humidified incubators in order for the virus particles to be produced.

24h and 48h after transfection, cell culture supernatant (now containing viral particles) was harvested, and replaced with fresh medium. Supernatants were filtered through a 45nm filter and stored at 4^oC for up to a week or at -80^oC long term.

2.2.4. Viral transduction of MS5 cells

Hif1a$^{-/-}$ MS5 cells were seeded in 6-well tissue culture plates at low density (10^5 cells per well) to be ~30% confluent the next day. After 24h, tissue culture medium was removed and substituted with 2ml of undiluted viral supernatant. Plates were then spun down at 400g for 90min, at room temperature, to increase viral transduction efficiency, and then transferred to the cell culture incubator. 24h after, viral supernatant was removed and fresh medium was added. Cells were then allowed to recover for 24h before adding blasticidin at a concentration of 50µg/ml (this optimal concentration had been previously determined by performing a kill curve with the *Hif1a*$^{-/-}$ MS5 cells) for selection of cells that had successfully integrated the transgenes. Cells were kept under selection for one week prior to performing any experiments with them.

2.3. Generation and characterization of Hif-1α mutant MS5 cell lines.

Once the desirable mutations had been introduced, three different versions of the *Hif1a* cDNA (WT cDNA, bHLH-mutated cDNA and PAS-A-mutated cDNA) were cloned into. Lentiviral particles were generated as described above and Hif-1α$^{-/-}$ MS5 cells were transduced with the different cDNA expression constructs and also an empty vector to be used as control for the subsequent experiments. After selection, expression of the Hif-1α proteins was assessed by western blot. As shown in Supplementary Figure 4 A, lentiviral transduction with the three Hif-1α constructs resulted in an over-expression of the Hif-1α proteins when compared with WT MS5 cells, in hypoxia. Interestingly, the recombinant proteins were regulated in the same fashion as the endogenous Hif-1α, showing high

protein levels in hypoxia (2% O_2) but very low levels in normoxia (21% O_2). The WT cDNA and PAS-A-mutated cDNA transduced cell lines showed the highest Hif-1α expression, while the levels of the bHLH-mutated Hif-1α protein were slightly lower, maybe due to an effect of the mutations on protein stability, although this was not confirmed.

Immunofluorescence staining was used to assess subcellular localization of the Hif-1α recombinant proteins in order to confirm their correct translocation to the nucleus, where Hif-1α performs its functions. In line with the previous western blot results, in hypoxia, the recombinant Hif-1α proteins were over-expressed when comparison with the WT MS5 cells, while their levels were extremely low in normoxia (Supplementary Figure 4 B). In addition, proper translocation to the nucleus in hypoxia was confirmed by co-localization of the Hif-1α signal with the DAPI used to stain the nuclei. Hif-1α staining revealed a homogeneous pan-nuclear staining and exclusion from the nucleolus, in line with the previous literature in the field (Taylor et al., 2016).

3. Immunofluorescence microscopy methods

Cells were cultured on glass coverslips in 21% or 5% O_2 for 48 hours prior to irradiation. All cultures were fixed in 4% paraformaldehyde (Sigma Aldrich) and permeabilized in 0.1% Triton®-X100 solution. Unspecific antibody binding was prevented by blocking with 5% FCS / 2% Goat Serum / PBS for 1h at 37°C. Cells were then stained with anti-Hif-1α primary antibody (Abcam) (1:50 dilution in blocking buffer) for 1h at 37°C and washed 3x with PBS before staining with FITC-conjugated goat anti-rabbit secondary antibody (1:200 dilution in blocking buffer) for 1h at 37°C. Cultures were washed 3x with PBS and nuclei were counterstained with DAPI-containing Vectashield antifade mounting medium (Vector Labs). All images were captured using 40X magnification on a Delta Vision integrated microscope system (Applied Precision) controlled by SoftWoRx software mounted on an IX71 Olympus microscope. Images were processed and analysed using Fiji (Schindelin et al., 2012).

4. Label-free proteomic extended methods

Protein isolation:

Protein was isolated with the addition of trichloroacetic acid (20%). After centrifugation at 14,000rpm for 10mins and aspiration, cell pellets were twice washed in ice-cold acetone with centrifugation repeated. Protein pellets were resuspended in buffer of 8M Urea in 50mM Ammonium Bicarbonate (NH_4HCO_3). Protein concentration was determined using the Bradford Assay.

In-solution digestion:

Cysteine of plasma protein samples were reduced using dithiothreitol followed by alkylation with iodoacetamide. Dithiothreitol, iodoacetamide and urea concentrations were diluted using 50mM NH_4HCO_3 before Trypsin Singles^{TM} proteomic grade (Sigma) was added, ensuring a urea

concentration lower than 2M. Digestion was carried out overnight at 37°C. After drying in vacuum centrifuge, peptides were acidified by trifluoroacetic acid (TFA), desalted with c18 STAGE tips (Rappsilber et al., 2007), and resuspended in 0.1% TFA.

Mass Spectrometry:

Peptide fractions were analyzed on a quadrupole Orbitrap (Q-Exactive, Thermo Scientific) mass spectrometer equipped with a reversed-phase NanoLC UltiMate 3000 HPLC system (Dionex LC Packings, now Thermo Scientific). Peptide samples were loaded onto C18 reversed phase columns (5 cm length, 75 µm inner diameter) and eluted with a linear gradient from 8 to 40% acetonitrile containing 0.5% TFA in 60 min at a flow rate of 3 µL/min. The injection volume was 5 µl. The mass spectrometer was operated in data dependent mode, automatically switching between MS and MS2 acquisition. Survey full scan MS spectra (m/z 350 – 1600) were acquired in the Orbitrap with a resolution of 70,000. MS2 spectra had a resolution of 17,500. The twelve most intense ions were sequentially isolated and fragmented by higher-energy C-trap dissociation.

Protein identification:

Raw data from the Orbitrap Q-Exactive was processed using MaxQuant version 1.5.1.0 (Cox and Mann, 2008; Tyanova et al., 2016a), incorporating the Andromeda search engine (Cox et al., 2011). To identify peptides and proteins, MS/MS spectra were matched to the Uniprot *mus musculus* database (2016_10) containing 50,306 entries. All searches were performed with tryptic specificity allowing two missed cleavages. The database searches were performed with carbamidomethyl (C) as fixed modification and acetylation (protein N terminus) and oxidation (M) as variable modifications. Mass spectra were searched using the default setting of MaxQuant namely a false discovery rate of 1% on the peptide and protein level. For the generation of label free quantitative (LFQ) ion intensities for protein profiles, signals of corresponding peptides in different nano-HPLC MS/MS runs were matched by MaxQuant applying a mass accuracy of at least 20 ppm and a maximum time window of 1 min (Cox et al., 2014).

Proteomic data analysis:

The Perseus statistical software (version 1.4.1.3) was used to analysis the LFQ intensities. Protein identifications were filtered to eliminate the identifications from the reverse database and common contaminants and the total number of proteins remaining was defined as the number of proteins identified. Data was log transformed and t-test comparison of fractions carried out. For tests derived from volcano plots, missing values were imputed with values from a normal distribution and a t-test comparison was performed. For visualization using heat maps, the dataset was normalized by z-score (Deeb et al., 2012; Tyanova et al., 2016b). The Metascape online tool (http://metascape.org) was used for GO term enrichment analysis (Tripathi et al., 2015).

5. Antibodies

For western blotting, anti-Hif-1α (EPR16897) rabbit monoclonal antibody (Abcam), anti-Hif-1β (Arnt) (2B10) mouse monoclonal antibody (Novus Biologicals), anti-phospho-Histone H2A.X (Ser139) mouse monoclonal antibody (Millipore), anti-H2AX rabbit polyclonal antibody (Abcam), anti-β-Actin rabbit polyclonal antibody (Sigma-Aldrich), Pierce horseradish peroxidase (HRP)-conjugated rabbit anti-mouse IgG antibody, Immuno-Pure HRP-conjugated goat anti-rabbit IgG antibody (Thermo Scientific), IRDye® Goat anti-Mouse and Goat anti-Rabbit IgG (LiCor) were used. For immunofluorescence staining, anti-Hif-1α (EPR16897) rabbit monoclonal antibody (Abcam) and fluorescein (FITC)-conjugated AffiniPure F(ab0)2 fragment goat anti-mouse IgG antibody were used.

SUPPLEMENTARY FIGURE LEGENDS

Supplementary Figure 1: ***Hif1a knockout CRISPR/Cas9 strategy***. **(A)** Mouse *Hif1a* genomic locus and cDNA structure. Untraslated regions are shown in white while translated regions are shown in gray. Two alternative ATG sites are indicated with green arrowheads, while stop codon (TGA) is indicated in red. **(B)** Hif-1α CRISPR/Cas9 knockoout strategy. Guide RNAs designed according to the two alternative first exons described in Wenger et al. 1998. Cas9 cut sites are indicated by red arrows. ATG in first exon is indicated in bold.

Supplementary Figure 2: ***Generation of Hif1a knockout MS5 cell line using CRISPR/Cas9 technology***. **(A)** Representative western blot showing three of the Hif-1α$^{-/-}$ MS5 cell lines obtained (Clones 41, 55 and 76). **(B)** Sequencing raw data corresponding to WT and mutated Hif1a exon 3. Specific mutations present in each allele of **(C)** exon 1 and **(D)** exon 3 of the *Hif1a* genomic locus of *Hif1a*$^{-/-}$ MS5 clones 41, 55 and 76. Cas9 cut sites are indicated with red arrows.

Supplementary Figure 3: ***Design strategy of MS5 cell lines containing WT and mutant Hif-1α cDNA***. Schematic representation of **(A)** Hif-1*α wild type* (WT) and mutant proteins with **(B)** defective interaction with DNA (Hif-1α$^{K19Q/R30Q}$) (bHLH-mutated) or **(C)** defective interaction with Arnt (Hif-1α$^{R170A/V191D}$) (PAS-A-mutated). Mutated nucleotides and amino acids are shown in red, while the original codons and amino acids are indicated in blue.

Supplementary Figure 4: ***Generation of MS5 cell lines containing WT and mutant Hif1a cDNA***. Representative **(A)** Hif-1α western blot and **(B)** immunofluorescence images showing the levels of expression and subcellular localization of the Hif-1α protein in WT, *Hif1a*$^{-/-}$, and *Hif1a*$^{-/-}$ MS5 transduced with the different versions of the *Hif1a* cDNA previously described (Figure 3.6) in normoxia (21% O_2) and hypoxia (2% O_2). Hif-1α is shown in green, while DAPI is shown in blue. Scale bars represent approximately 20μm.

Supplementary Figure 5: ***Heat maps comparing effect of hypoxia on the proteome of WT and* Hif1a$^{-/-}$ *MS5 cells***. Heat maps with hierarchical clustering comparing the significant proteins ($p<0.05$) obtained trough a Log_2 transformed intensity t-test in **(A)** WT MS5 and **(B)** *Hif1a*$^{-/-}$ MS5 samples. Intensity color code represents ion current intensity (Log_2 transformed, z-score normalized).

Supplementary Figure 6: ***Full X-ray film images corresponding to western blots shown in Figure 2***. Original un-cropped images of the X-ray films used to produce the western blots depicted in **(A)** Figure 2C, **(B)** Figure 2E and **(C)** Figure 2G. When necessary, films corresponding to the same western blot but different exposure times are shown.

Supplementary Figure 7: ***Full LiCor scan images corresponding to western blots shown in Figure 5***. Original un-cropped LiCor scans used to produce the western blots depicted in Figure 5: **(A)** WT MS5, **(B)** *Hif1a*$^{-/-}$ MS5 clone 41, **(C)** *Hif1a*$^{-/-}$ MS5 Clone 41 transduced with and empty lentiviral vector, **(D)** WT *Hif1a* cDNA, **(E)** bHLH-mutated Hif-1α cDNA, and **(F)** PAS-A-mutated *Hif1a* cDNA lentiviral constructs.

Supplementary Figure 8: ***Full X-ray film images corresponding to western blots shown in Supplementary Figure 2***. Original un-cropped images of the X-ray films used to produce the western blots depicted in Supplementary Figure 2A. Films corresponding to the same western blot but different exposure times are shown. Note that after the transfer, the membrane was cut and different pieces probed with antibodies against β-Actin and Hif-1α.

Supplementary Figure 9: ***Full X-ray film images corresponding to western blots shown in Supplementary Figure 4***. Original un-cropped images of the X-ray films used to produce the western blots depicted in Supplementary Figure 4A. Note that after the transfer, the membrane was cut and different pieces probed with antibodies against β-Actin and Hif-1α.

SUPPLEMENTARY TABLES

Supplementary Table 1: ***Guide RNA sequences designed for Hif1a knockout***. Overhangs for cloning are shown in blue.

Name	Sequence (5' to 3')	Exon Targeted
Guide RNA 1 Forward	CACCGGGCACCGATTCGCCATGGA	Exon 1.2
Guide RNA 1 Reverse	AAACTCCATGGCGAATCGGTGCCC	Exon 1.2
Guide RNA 2 Forward	CACCGCTCGCTCGGGCCTAAACGC	Exon 1.2
Guide RNA 2 Reverse	AAACGCGTTTAGGCCCGAGCGAGC	Exon 1.2
Guide RNA 3 Forward	CACCGCTAACAGATGACGGCGACA	Exon 3
Guide RNA 3 Reverse	AAACTGTCGCCGTCATCTGTTAGC	Exon 3

Supplementary Table 2: ***Primer sequences***. Cloning overhangs are shown in blue.

Primer	Sequence (5' to 3')	Use
CRISPR Exon 1 Forward Primer	CGCGGGCAGTGTCTAGCCAG	PCR and sequencing of *Hif1a* exon 1.2
CRISPR Exon 1 Reverse Primer	GGCCCGGCTTACTTTTTCTTC	PCR and sequencing of *Hif1a* exon 1.2
CRISPR Exon 3 Forward Primer	CGTGTGCCCTGCAGGTGGTC	PCR and sequencing of *Hif1a* exon 3
CRISPR Exon 3 Reverse Primer	CAATATCTGACTGAAAATCACCTGTC	PCR and sequencing of *Hif1a* exon 3
Hif1a cDNA Forward Primer	GGGGACAAGTTTGTACAAAAAAGCA GGCTACATGGAGGGCGCCGGCGGCG	*Hif1a* cDNA Isolation
Hif1a cDNA Reverse Primer	GGGGACCACTTTGTACAAGAAAGCTGGGTA GTTAACTTGATCCAAAGCTCTGAGTAATTC	*Hif1a* cDNA Isolation
*Hif1a*_Seq1	GTGAAGATGAGATGAAGGCAC	*Hif1a* cDNA Sequencing
*Hif1a*_Seq2	GATAGCAAGACATTTCTCAGTCG	*Hif1a* cDNA Sequencing
*Hif1a*_Seq3	CTGCCGGCGACACCATCATC	*Hif1a* cDNA Sequencing
*Hif1a*_Seq4	GAGATGCTGGCTCCCTATATC	*Hif1a* cDNA Sequencing
Hif1a bHLH Forward Primer	GAGTTCTGAACGTCGACAAGAAAA GTCTAGAGATGCAGCAAGATCTCG	Site-directed mutagenesis of bHLH
Hif1a bHLH Reverse Primer	CTTCAGACTCTTTGCTTTGCCGA GATCTTGCTGCATCTCTAGACTT	Site-directed mutagenesis of bHLH
Hif1a PAS-A Forward Primer	GCGGAGCTTTTTTCTCGCAATGAAGTGCA CCCTAACAAGCCGGGGGAGGACGATGAA	Site-directed mutagenesis of PAS-A

Hif1a PAS-A Reverse Primer	GGCCCGTGCAGTGAAGGTCCTTCCACGTT GCTGACTTGATGTTCATCGTCCTCCCCCG	Site-directed mutagenesis of PAS-A
pX330_seq	AGGGATGGTTGGTTGGTGGG	Sequencing of pX330
pDONR201_seq	GTAACATCAGAGATTTTGAGACAC	Sequencing of pDONR201
hPGK_seq	GTGTTCCGCATTCTGCAAG	Sequencing of pLENTI PGK

Supplementary Table 3: ***Results of WT MS5 proteomic analysis***. Complete list of proteins displaying significantly ($p<0.05$) different levels in hypoxic (2% O_2) compared to normoxic (21% O_2) WT MS5 cells.

Protein Name	Description	Fold Change (2% / 21%)	p Value
Fam162a	Protein FAM162A	8.828432064	0.036140319
Pdk1	Pyruvate Dehydrogenase Kinase 1	6.746427255	0.021028868
Clip1	CAP-Gly domain-containing linker protein 1	6.591873249	0.002062491
Pgrmc2	Membrane-associated progesterone receptor component 2	5.718281992	0.044519899
Ubl4	Ubiquitin-like protein 4A	5.102159199	0.017456936
Iap	IgE-binding protein	4.86250635	0.005688261
Esyt2	Extended synaptotagmin-2	4.241327506	0.008682936
Marc2	Mitochondrial amidoxime reducing component 2	3.966077475	0.002818987
Erlin2	Erlin-2	3.870047442	0.004848532
Spcs3	Signal peptidase complex subunit 3	3.687799871	0.036506442
Vapa	Vesicle-associated membrane protein-associated protein A	3.523118999	0.00591052
Uchl5	Ubiquitin carboxyl-terminal hydrolase isozyme L5	3.509154652	0.033818988
Plod2	Procollagen-lysine,2-oxoglutarate 5-dioxygenase 2	3.42425442	0.045033327
Hk2	Hexokinase	3.393273016	0.041917218
Pfkl	ATP-dependent 6-phosphofructokinase, liver type	3.271238528	0.007701574
Tmed2	Transmembrane emp24 domain-containing protein 2	3.231685508	0.013515208
Pgm1	Phosphoglucomutase-1	3.118091246	0.012470469
Rabgap1	Rab GTPase-activating protein 1	3.008654246	0.013988122
Krr1	KRR1 small subunit processome component homolog	2.826301458	0.033109432
Ints4	Integrator complex subunit 4	2.619363178	0.015185208
Fam91a1	Protein FAM91A1	2.541265938	0.031218297
Cyb5r3	NADH-cytochrome b5 reductase	2.458804622	0.045039282
Reep5	Receptor expression-enhancing protein	2.398783632	0.035452085
Plxnb2	Plexin-B2	2.378572505	0.049215033
Cox4i1	Cytochrome c oxidase subunit 4 isoform 1, mitochondrial	2.320120445	0.019966959
Stt3a	Dolichyl-diphosphooligosaccharide--protein glycosyltransferase subunit STT3A	2.29382678	0.0144006
Atp2a2	Calcium-transporting ATPase	2.263109797	0.04375561
Ldha	L-lactate dehydrogenase	2.252571661	0.000562825
Rpn2	Dolichyl-diphosphooligosaccharide--protein glycosyltransferase subunit 2	2.218297475	0.046067601
mt-Co2	Cytochrome c oxidase subunit 2	2.201658173	0.035370894
Mtch2	Mitochondrial carrier homolog 2	2.150586217	0.048589965
P4ha2	Prolyl 4-hydroxylase subunit alpha-2	2.133186272	0.022184707
Pgk1	Phosphoglycerate kinase 1	2.130788546	0.001792476
Glg1	Golgi apparatus protein 1	2.114910958	0.021158687
Gpi	Glucose-6-phosphate isomerase	2.103769188	0.015255237
Surf4	Surfeit locus protein 4	2.086434466	0.012152539
Cav1	Caveolin-1	2.013809358	0.015071321

Cisd2	CDGSH iron-sulfur domain-containing protein 2	2.007387254	0.003186504
Ptgs1	Prostaglandin G/H synthase 1	2.00012516	0.016824688
Ddx54	ATP-dependent RNA helicase DDX54	1.998048691	0.020696409
Ralb	Ras-related protein Ral-B	1.919773052	0.006493314
Tpi1	Triosephosphate isomerase	1.894096797	0.003997836
Hibch	3-hydroxyisobutyryl-CoA hydrolase, mitochondrial	1.880475935	0.049307223
Tinagl1	Tubulointerstitial nephritis antigen-like	1.842834125	0.014960471
Prpf6	Pre-mRNA-processing factor 6	1.793120045	0.013836782
Pgam1	Phosphoglycerate mutase 1	1.788359816	0.005171179
Atl3	Atlastin-3	1.77949273	0.011197487
Rtn4	Reticulon	1.768301368	0.009592604
Sec11a	Signal peptidase complex catalytic subunit SEC11	1.763689411	0.029563685
Rac1	Ras-related C3 botulinum toxin substrate 1	1.745922366	0.024397149
Mtx1	Metaxin-1	1.736333448	0.031002631
Cdc42	Cell division control protein 42 homolog	1.7338797	0.015773976
Atp1a1	Sodium/potassium-transporting ATPase subunit alpha-1	1.702943024	0.032392691
Slc25a5	ADP/ATP translocase 2	1.680639254	0.034307817
Galk1	Galactokinase	1.663189828	0.028386352
Sfxn3	Sideroflexin-3	1.662104679	0.041464289
Fbln2	Fibulin-2	1.643615874	0.032346603
Aldoa	Fructose-bisphosphate aldolase	1.639395578	0.017903381
Slc25a4	ADP/ATP translocase 1	1.638580838	0.03363805
Map2k1	Dual specificity mitogen-activated protein kinase kinase 1	1.636454166	0.017966329
Itgb1	Integrin beta-1	1.635142882	0.006902341
Rab5c	Ras-related protein Rab-5C	1.569288802	0.001171308
Rtn4	Reticulon-4	1.5624124	0.036687995
Rras2	Ras-related protein R-Ras2	1.558944629	0.019240147
P4ha1	Prolyl 4-hydroxylase subunit alpha-1	1.541445063	0.008690228
Mrpl39	39S ribosomal protein L39, mitochondrial	1.521486429	0.009448518
Fermt2	Fermitin family homolog 2	1.521280597	0.041672094
Rhoa	Transforming protein RhoA	1.512054282	0.002178632
Rap1b	Ras-related protein Rap-1b	1.509639366	0.007731565
Tmed10	Transmembrane emp24 domain-containing protein 10	1.504193215	0.027878579
Slc25a24	Calcium-binding mitochondrial carrier protein SCaMC-1	1.499329555	0.002397084
Myof	Myoferlin	1.480481787	0.028383907
Plbd2	Putative phospholipase B-like 2	1.445452143	0.009831839
Rab2a	Ras-related protein Rab-2A	1.414085806	0.017532373
Rab6a	Ras-related protein Rab-6A	1.403338448	0.017861604
Eno1	Alpha-enolase	1.381660048	0.026443296
Mif	Macrophage migration inhibitory factor	1.361381022	0.020445282
Srpr	Signal recognition particle receptor subunit alpha	1.357836947	0.013641844
Nras	GTPase KRas	1.351648217	0.026448487
Pkm	Pyruvate kinase PKM	1.347727923	0.021963784
Rab11b	Ras-related protein Rab-11B	1.333588641	0.024422138
Mthfd1l	Monofunctional C1-tetrahydrofolate synthase,	1.316941015	0.042333773
Csrp1	Cysteine and glycine-rich protein 1	1.311862778	0.033458329
Gapdh	Glyceraldehyde-3-phosphate dehydrogenase	1.298644482	0.016005042
Gnas	Guanine nucleotide-binding protein G(s) subunit alpha	1.291218441	0.031191002
Anxa2	Annexin	1.253653639	0.003676734
Anxa5	Annexin A5	1.251204156	0.044965402
Samm50	Sorting and assembly machinery component 50 homolog	1.241032248	0.002922139
Rnf113a2	Protein Rnf113a2	1.223666833	0.018161335
Cycs	Cytochrome c, somatic	1.203269784	0.021364371
Flna	Filamin-A	1.129688934	0.020981037
Ehd1	EH domain-containing protein 1	1.068430686	0.023105893
Esd	S-formylglutathione hydrolase	0.882935851	0.027071083
Cdc37	Hsp90 co-chaperone Cdc37	0.879289733	0.024551989
Ilf3	Interleukin enhancer-binding factor 3	0.871388	0.042853436

Pea15	Astrocytic phosphoprotein PEA-15	0.866158522	0.035042291
Psmc6	26S protease regulatory subunit 10B	0.861104239	0.032700617
Ddx1	ATP-dependent RNA helicase DDX1	0.856710095	0.016180137
Eif3i	Eukaryotic translation initiation factor 3 subunit I	0.852357529	0.007349531
Pnp	Purine nucleoside phosphorylase	0.852270389	0.020416218
Psma3	Proteasome subunit alpha type	0.852201283	0.048706293
Ranbp3	Ran-binding protein 3	0.85118638	0.028371174
Myg1	UPF0160 protein MYG1, mitochondrial	0.841753252	0.039878466
Gpx7	Glutathione peroxidase	0.841430955	0.034847111
Hsd17b4	Peroxisomal multifunctional enzyme type 2	0.837180735	0.023325883
Oat	Ornithine aminotransferase, mitochondrial	0.825749731	0.027127987
Pdia3	Protein disulfide-isomerase A3	0.825501588	0.027220448
Hnrnpu	Heterogeneous nuclear ribonucleoprotein U	0.82268774	0.013962073
Sept2	Septin-2	0.81378344	0.032041881
Psmc5	26S protease regulatory subunit 8	0.812778112	0.048803075
Rpl9	60S ribosomal protein L9	0.810439318	0.019772828
Lap3	Cytosol aminopeptidase	0.807445487	0.035254437
Ndufs5	NADH dehydrogenase [ubiquinone] iron-sulfur protein 5	0.807037804	0.029998858
Hsd17b10	3-hydroxyacyl-CoA dehydrogenase type-2	0.805565317	0.043805514
Ddt	D-dopachrome decarboxylase	0.803531578	0.037971635
Ahsa1	Activator of 90 kDa heat shock protein ATPase homolog 1	0.802113914	0.046741495
Dcps	m7GpppX diphosphatase	0.800198904	0.026920874
Eif3h	Eukaryotic translation initiation factor 3 subunit H	0.792555484	0.004873818
Fkbp10	Peptidyl-prolyl cis-trans isomerase FKBP10	0.789242248	0.004226024
Rps18	40S ribosomal protein S18	0.786973259	0.045723225
Fkbp3	Peptidyl-prolyl cis-trans isomerase	0.784736039	0.013963111
Luc7l2	Putative RNA-binding protein Luc7-like 2	0.779729987	0.019337176
Pdia6	Protein disulfide-isomerase A6	0.777387961	0.003874364
Tmem167	Protein kish	0.775334465	0.03583713
Ube2n	Ubiquitin-conjugating enzyme E2 N	0.772594577	0.043310956
Pcolce	Procollagen C-endopeptidase enhancer 1	0.767506317	0.025054629
Ctsd	Cathepsin D	0.759650741	0.036483141
Phgdh	D-3-phosphoglycerate dehydrogenase	0.759399034	0.023116157
Rrp9	U3 small nucleolar RNA-interacting protein 2	0.755265555	0.031644586
Gfm1	Elongation factor G, mitochondrial	0.754341827	0.004046929
Hyou1	Hypoxia up-regulated protein 1	0.753514527	0.012798813
Rps16	40S ribosomal protein S16	0.749891446	0.036673267
Pcna	Proliferating cell nuclear antigen	0.741190795	0.018871559
Oxct1	Succinyl-CoA:3-ketoacid coenzyme A transferase 1,	0.725519596	0.037890441
Prkar1a	cAMP-dependent protein kinase type I-alpha regulatory	0.720784566	0.003194986
Ppid	Peptidyl-prolyl cis-trans isomerase D	0.720593053	0.042435077
Prkcsh	Glucosidase 2 subunit beta	0.716267281	0.038460653
Idh3b	Isocitrate dehydrogenase [NAD] subunit, mitochondrial	0.713523845	0.005331437
Lrrc40	Leucine-rich repeat-containing protein 40	0.713464418	0.000547686
Hsph1	Heat shock protein 105 kDa	0.703941018	0.015492007
Rpl21	60S ribosomal protein L21	0.690463268	0.034499312
Rps6	40S ribosomal protein S6	0.68447346	0.016932685
Sqstm1	Sequestosome-1	0.679718988	0.020679493
Rpl7a	60S ribosomal protein L7a	0.670125473	0.017548049
Ybx1	Nuclease-sensitive element-binding protein 1	0.668663767	0.048132357
Dhfr	Dihydrofolate reductase	0.634804051	0.042913772
Rpl34	60S ribosomal protein L34	0.622336176	0.025367293
Nolc1	Protein Nolc1	0.619020757	0.007211699
Ddx18	ATP-dependent RNA helicase DDX18	0.605314242	0.030534108
Plcg1	Phosphoinositide phospholipase C	0.60029639	0.046582461
Ftl1	Ferritin	0.597575905	0.017505635
Tmem14c	Transmembrane protein 14C	0.594890557	0.043060484

Fth1	Ferritin heavy chain	0.592270258	0.006472939
Uba5	Ubiquitin-like modifier-activating enzyme 5	0.589959634	0.021415064
Rpl24	60S ribosomal protein L24	0.585444079	0.008237576
Rpl27	60S ribosomal protein L27	0.57168788	0.037981037
Rpl13	60S ribosomal protein L13	0.56263588	0.009392417
H2afv	Histone H2A	0.562352546	0.021939871
Tbc1d5	TBC1 domain family member 5	0.547362621	0.01336319
Cul2	Cullin-2	0.544332898	0.021807055
Rpl26	60S ribosomal protein L26	0.539147978	0.03180696
Brox	BRO1 domain-containing protein BROX	0.536940238	0.046242166
Aldh16a1	Aldehyde dehydrogenase family 16 member A1	0.525902896	0.021733951
Luzp4	Leucine Zipper Protein 4	0.516723717	0.018301366
Renbp	N-acylglucosamine 2-epimerase	0.509118297	0.045757454
Rpl35	60S ribosomal protein L35	0.450886766	0.036036982
Nudt3	Diphosphoinositol polyphosphate phosphohydrolase 1	0.449446496	0.001198448
Map6	Microtubule-associated protein 6	0.443419292	0.030740301
Nup85	Nuclear pore complex protein Nup85	0.44297066	0.039814933
Col3a1	Collagen alpha-1(III) chain	0.435664816	0.030648012
Abhd12	Monoacylglycerol lipase ABHD12	0.435273325	0.014415502
Tor1aip2	Torsin-1A-interacting protein 2, isoform IFRG15	0.348065868	0.019190008
Prps1l1	Protein Prps1l1	0.311767095	0.036305065

Supplementary Table 4: ***Results of* Hif1a$^{-/-}$ *MS5 proteomic analysis***. Complete list of proteins displaying significantly ($p<0.05$) different levels in hypoxic (2% O_2) compared to normoxic (21% O_2) *Hif1a$^{-/-}$* MS5 cells.

Protein Name	Description	Fold Change (2% / 21%)	p Value
Cops3	COP9 signalosome complex subunit 3	4.147513651	0.000574502
Stt3b	Dolichyl-diphosphooligosaccharide-protein glycosyltransferase subunit STT3B	3.993199753	0.021713159
Pgm2	Phosphoglucomutase-2	3.011538106	0.016277116
Ccar2	Cell cycle and apoptosis regulator protein 2	2.01044689	0.002115818
Ano6	Anoctamin-6	1.999500319	0.03500315
Gsk3b	Glycogen synthase kinase-3 beta	1.935081709	0.015039603
Glg1	Golgi apparatus protein 1	1.905535865	0.011893252
Rpia	Ribose-5-phosphate isomerase	1.726136025	0.031395245
Slc25a3	Phosphate carrier protein, mitochondrial	1.704565566	0.045039935
Dync1h1	Cytoplasmic dynein 1 heavy chain 1	1.668130659	0.031447981
mt-Co2	Cytochrome c oxidase subunit 2	1.631809939	0.048653748
Tnpo1	Transportin-1	1.602834997	0.002307713
Rab21	Ras-related protein Rab-21	1.594005246	0.043798748
Myof	Myoferlin	1.565727106	0.036743851
Pgm1	Phosphoglucomutase-1	1.551083105	0.003574611
Fbln2	Fibulin-2	1.535004516	0.040289398
Rbm22	Pre-mRNA-splicing factor RBM22	1.528297201	0.037071344
Ikbkap	Elongator complex protein 1	1.528203587	0.014526543
Bax	Apoptosis regulator BAX	1.510283499	0.015801213
Myh9	Myosin-9	1.48347165	0.015153221
Cltc	Clathrin heavy chain	1.472968552	0.025216626
Flii	Protein flightless-1 homolog	1.471216959	0.011434549
Aco1	Cytoplasmic aconitate hydratase	1.468733922	0.021099568
Arf3	ADP-ribosylation factor 1	1.465574822	0.001095955
Ezr	Ezrin	1.46104785	0.036478713
Rab2a	Ras-related protein Rab-2A	1.440955576	0.040840757
Rars	Arginine--tRNA ligase, cytoplasmic	1.417045241	0.033783281
Ube3a	Ubiquitin-protein ligase E3A	1.393187182	0.008304521
Got1	Aspartate aminotransferase, cytoplasmic	1.383517177	0.049540979

Capn1	Calpain-1 catalytic subunit	1.382749773	0.044475106
Ehd4	EH domain-containing protein 4	1.369105592	0.019981748
Ywhah	14-3-3 protein eta	1.367009342	0.035415604
Prkacb	cAMP-dependent protein kinase catalytic subunit beta	1.346972653	0.032956991
Csnk2a1	Casein kinase II subunit alpha	1.341152561	0.033659991
Iqgap1	Ras GTPase-activating-like protein IQGAP1	1.302629793	0.038369903
Calm1	Calmodulin	1.291968069	0.042294001
Ywhag	14-3-3 protein gamma	1.287459231	0.030820116
Pls3	Plastin-3	1.28661696	0.017799192
Actn1	Alpha-actinin-1	1.282928248	0.018303297
Anxa5	Annexin A5	1.281328105	0.017400716
Cand1	Cullin-associated NEDD8-dissociated protein 1	1.271507362	0.045129179
Sec24d	SEC24 Homolog D, COPII Coat Complex Component	1.268593565	0.032668396
Prep	Prolyl endopeptidase	1.26233753	0.032381351
Tbc1d15	TBC1 domain family member 15	1.260829188	0.014936573
Pygb	Glycogen phosphorylase, brain form	1.259193346	0.045768911
Tra2a	Transformer-2 protein homolog alpha	1.25055368	0.025377909
Otub1	Ubiquitin thioesterase OTUB1	1.249380921	0.010488924
Flna	Filamin-A	1.246710659	0.03388551
Prkcd	Protein kinase C delta type	1.246639237	0.030967486
Myl12a	Myosin Light Chain 12A	1.238038787	0.036112036
Psmd1	26S proteasome non-ATPase regulatory subunit 1	1.229049659	0.031626553
Ap2a2	AP-2 complex subunit alpha-2	1.221344833	0.038960661
Txnl1	Thioredoxin-like protein 1	1.221121485	0.031806109
Psmd13	26S proteasome non-ATPase regulatory subunit 13	1.210172949	0.016459105
Eftud2	116 kDa U5 small nuclear ribonucleoprotein component	1.205482513	0.032880992
Psmb3	Proteasome subunit beta type	1.198716505	0.037274992
Ywhae	14-3-3 protein epsilon	1.155560959	0.029929464
Nedd4	E3 ubiquitin-protein ligase NEDD4	1.153261969	0.042894485
Ppp1ca	Serine/threonine-protein phosphatase PP1-alpha catalytic	1.131108181	0.001877598
Tuba1a	Tubulin alpha-1A chain	1.113825065	0.036696287
Gpi	Glucose-6-phosphate isomerase	1.104497398	0.035821167
Igfbp7	Insulin-like growth factor-binding protein 7	0.924498517	0.038533126
Gnb2l1	Guanine nucleotide-binding protein subunit beta-2-like 1	0.899868804	0.020717633
Psmd5	26S proteasome non-ATPase regulatory subunit 5	0.897987095	0.016993004
Cbx5	Chromobox protein homolog 5	0.89117673	0.032837012
Epb41l2	Band 4.1-like protein 2	0.885396842	0.04945504
Ctps1	CTP synthase 1	0.883040915	0.038684581
Luc7l2	Putative RNA-binding protein Luc7-like 2	0.86880857	0.048704593
Prpf19	Pre-mRNA-processing factor 19	0.868507299	0.017419104
Rps3	40S ribosomal protein S3	0.857661279	0.001247683
Calr	Calreticulin	0.849203984	0.020961262
Ola1	Obg-like ATPase 1	0.848116027	0.02237107
Smc2	Structural maintenance of chromosomes protein 2	0.839986034	0.007498138
Akr1b3	Aldose reductase	0.831518691	0.01868705
Eef1a1	Elongation factor 1-alpha	0.827265675	0.003707584
St13	Hsc70-interacting protein	0.824021195	0.009975812
Matr3	Matrin-3	0.821895223	0.02421145
Bub3	Mitotic checkpoint protein BUB3	0.820990217	0.046905706
Fkbp9	Peptidyl-prolyl cis-trans isomerase FKBP9	0.816895804	0.020026678
Dlst	Dihydrolipoyllysine-residue succinyltransferase component of 2-oxoglutarate dehydrogenase complex, mitochondrial	0.815011947	0.006406037
Txndc5	Thioredoxin domain-containing protein 5	0.814955559	0.005940789
Pabpc4	Polyadenylate-binding protein	0.813691278	0.021725697
Cisd1	CDGSH iron-sulfur domain-containing protein 1	0.797562332	0.045738059
Aimp1	Aminoacyl tRNA synthase complex-interacting multifunctional protein 1	0.793799169	0.016749441
Umps	Uridine 5-monophosphate synthase	0.791907151	0.044313294

Dkc1	H/ACA ribonucleoprotein complex subunit 4	0.775482769	0.027470028
Prkar1a	cAMP-dependent protein kinase type I-alpha regulatory subunit	0.773695097	0.013140989
Gtf2i	General transcription factor II-I	0.771713589	0.043976078
Rbmxl1	RNA binding motif protein, X-linked-like-1	0.770544575	0.011008225
Mcm4	DNA replication licensing factor MCM4	0.770115475	0.001866342
Hat1	Histone acetyltransferase type B catalytic subunit	0.764912739	0.016077125
Dnaja1	DnaJ homolog subfamily A member 1	0.757382376	0.038676627
Hnrnpul1	Heterogeneous nuclear ribonucleoprotein U-like protein 1	0.756652102	0.010054471
Hnrnpab	Heterogeneous nuclear ribonucleoprotein A/B	0.754944767	0.010319356
Psmd7	26S proteasome non-ATPase regulatory subunit 7	0.752467251	0.000463265
Rbm17	Splicing factor 45	0.740728423	0.00805526
Abhd14b	Alpha/beta hydrolase domain-containing protein 14B	0.739538554	0.039266265
Gfm1	Elongation factor G, mitochondrial	0.738513632	0.000603664
Rprd1b	Regulation of nuclear pre-mRNA domain-containing protein 1B	0.73269635	0.016471582
Mcm5	DNA helicase	0.731461358	0.003310837
Ddx5	Probable ATP-dependent RNA helicase DDX5	0.723490554	0.002544133
Chordc1	Cysteine and histidine-rich domain-containing protein 1	0.705057766	0.0103453
Rrp9	U3 small nucleolar RNA-interacting protein 2	0.702769361	0.0301801
Srrm1	Serine/arginine repetitive matrix protein 1	0.701277244	0.046264502
Hspe1	10 kDa heat shock protein, mitochondrial	0.697584314	0.001823468
Ddx1	ATP-dependent RNA helicase DDX1	0.697583391	0.003855206
Txndc12	Thioredoxin domain-containing protein 12	0.696122839	0.008830506
Txndc9	Thioredoxin domain-containing protein 9	0.69145289	0.027398734
Sparc	Secreted Protein Acidic And Cysteine Rich	0.686473846	0.008774403
Rpl31	60S ribosomal protein L31	0.681360683	0.011397697
Larp4	La-related protein 4	0.676631411	0.025737877
Rps14	40S ribosomal protein S14	0.67340946	0.022451426
Parva	Alpha-parvin	0.66996602	0.025654396
Smu1	WD40 repeat-containing protein SMU1	0.668980617	0.003873481
Nip7	60S ribosome subunit biogenesis protein NIP7 homolog	0.667192018	0.036100876
Serpinh1	Serpin H1	0.659820947	0.008657068
Hnrnpc	Heterogeneous nuclear ribonucleoproteins C1/C2	0.659369815	0.012641919
Rpl30	60S ribosomal protein L30	0.658242464	0.028693544
Ehd2	EH domain-containing protein 2	0.658199823	0.043021931
Rps6	40S ribosomal protein S6	0.652767156	0.022151816
Mta2	Metastasis-associated protein MTA2	0.65187944	0.044864374
Cnpy2	Protein canopy homolog 2	0.647977583	0.002885027
Rnps1	RNA-binding protein with serine-rich domain 1	0.646839778	0.044810766
Fen1	Flap endonuclease 1	0.6460945	0.017656999
Btf3	Transcription factor BTF3	0.636545937	0.017066934
Chd4	Chromodomain-helicase-DNA-binding protein 4	0.636310625	0.004910422
Rps15a	40S ribosomal protein S15a	0.631478979	0.019893563
Rps2	40S ribosomal protein S2	0.628052299	0.038181555
Plrg1	Pleiotropic regulator 1	0.625979346	0.004958462
Alyref	THO complex subunit 4	0.624224284	0.01453206
Fbl	rRNA 2-O-methyltransferase fibrillarin	0.616216501	0.024913818
Rps20	40S ribosomal protein S20	0.613500376	0.02401769
Rfc1	Replication factor C subunit 1	0.60444524	0.003938612
Rps29	40S ribosomal protein S29	0.601115187	0.003266851
Ddx18	ATP-dependent RNA helicase DDX18	0.598930512	0.000980768
Gulp1	PTB domain-containing engulfment adapter protein 1	0.598216707	0.024361435
Cdc5l	Cell division cycle 5-like protein	0.595443453	0.007063314
Rps11	40S ribosomal protein S11	0.584040438	0.015348209
Rps10	40S ribosomal protein S10	0.583827108	0.001316875
Rpl8	60S ribosomal protein L8	0.581796276	0.041303366
Sh3glb2	Endophilin-B2	0.579569422	0.035026718
Racgap1	Rac GTPase-activating protein 1	0.565631857	0.020601459

Nolc1	Nucleolar And Coiled-Body Phosphoprotein 1	0.561098471	0.018819857
Krt2	Keratin, type II cytoskeletal 2 epidermal	0.556644346	0.019408957
Rpl23	60S ribosomal protein L23	0.552460472	0.004570924
Fam3c	Protein FAM3C	0.540802052	0.019648027
Top2a	DNA topoisomerase 2-alpha	0.528587023	0.029008446
Rps25	40S ribosomal protein S25	0.521748395	0.040242251
Rpl13	60S ribosomal protein L13	0.517869255	0.009036138
Krt5	Keratin, type II cytoskeletal 5	0.511846216	0.006105927
Maged2	MAGE Family Member D2	0.490466269	0.018203496
Rpl38	60S ribosomal protein L38	0.48475147	0.031765135
Hist1h2bm	Histone H2B	0.482024972	0.005125118
Rpl35a	60S ribosomal protein L35a	0.477766312	0.028067478
Rps16	40S ribosomal protein S16	0.476309024	0.000829324
Phf5a	PHD finger-like domain-containing protein 5A	0.469733171	0.019126651
Lsm4	U6 snRNA-associated Sm-like protein LSm4	0.464069715	0.031705944
Rpl28	60S ribosomal protein L28	0.446633278	0.018267121
Rps19	40S ribosomal protein S19	0.43777049	0.003487514
Tmpo	Lamina-associated polypeptide 2, isoforms beta/delta/epsilon/gamma	0.432667403	0.015942676
Rpl34	60S ribosomal protein L34	0.427978235	0.013747715
Thop1	Thimet oligopeptidase	0.425734255	0.047262877
H2afv	Histone H2A	0.383782417	0.003820519
Nxf1	Nuclear RNA export factor 1	0.379597292	0.011584727
Rpl24	60S ribosomal protein L24	0.354184508	0.002932656
Fkbp2	Peptidyl-prolyl cis-trans isomerase	0.344066231	0.043616839
Arfgap1	ADP-ribosylation factor GTPase-activating protein 1	0.322174884	0.032749509
Hist1h2ah	Histone H2A	0.284882152	0.011073939
Dnajb11	DnaJ homolog subfamily B member 11	0.258660452	0.036565837
Glipr2	Golgi-associated plant pathogenesis-related protein 1	0.24113668	0.008931122
H2afy	Core histone macro-H2A.1	0.23031346	0.019313628
Hist2h3b	Histone H3	0.216614745	0.000256479
Hist2h4	Histone H4	0.20002328	0.000888762
Ufd1l	Ubiquitin fusion degradation protein 1 homolog	0.189151022	0.000903742
Hist1h1c	Histone H1.2	0.168290946	0.028303979

SUPPLEMENTARY REFERENCES

Cong, L., Ran, F. A., Cox, D., Lin, S., Barretto, R., Hsu, P. D., et al. (2013). Multiplex Genome Engineering Using CRISPR/Cas Systems. *Science (80-.).* 339, 819–823. doi:10.1126/science.1231143.Multiplex.

Cox, J., Hein, M. Y., Luber, C. a, and Paron, I. (2014). Accurate proteome-wide label-free quantification by delayed normalization and maximal peptide ratio extraction, termed MaxLFQ. *Mol. Cell. …* 13, 2513–2526. doi:10.1074/mcp.M113.031591.

Cox, J., and Mann, M. (2008). MaxQuant enables high peptide identification rates, individualized p.p.b.-range mass accuracies and proteome-wide protein quantification. *Nat. Biotechnol.* 26, 1367–72. doi:10.1038/nbt.1511.

Cox, J., Neuhauser, N., Michalski, A., Scheltema, R. A., Olsen, J. V., and Mann, M. (2011). Andromeda: A peptide search engine integrated into the MaxQuant environment. *J. Proteome Res.* 10, 1794–1805. doi:10.1021/pr101065j.

Deeb, S. J., D'Souza, R. C. J., Cox, J., Schmidt-Supprian, M., and Mann, M. (2012). Super-SILAC Allows Classification of Diffuse Large B-cell Lymphoma Subtypes by Their Protein Expression Profiles. *Mol. Cell. Proteomics* 11, 77–89. doi:10.1074/mcp.M111.015362.

Forbes, S. A., Beare, D., Gunasekaran, P., Leung, K., Bindal, N., Boutselakis, H., et al. (2015). COSMIC: exploring the world's knowledge of somatic mutations in human cancer. *Nucleic Acids Res.* 43, D805–D811. doi:10.1093/nar/gku1075.

Kewley, R. J., Whitelaw, M. L., and Chapman-Smith, A. (2004). The mammalian basic helix-loop-helix/PAS family of transcriptional regulators. *Int. J. Biochem. Cell Biol.* 36, 189–204. doi:10.1016/S1357-2725(03)00211-5.

Rappsilber, J., Mann, M., and Ishihama, Y. (2007). Protocol for micro-purification, enrichment, pre-fractionation and storage of peptides for proteomics using StageTips. *Nat. Protoc.* 2, 1896–1906. doi:10.1038/nprot.2007.261.

Schindelin, J., Arganda-Carreras, I., Frise, E., Kaynig, V., Longair, M., Pietzsch, T., et al. (2012). Fiji: an open-source platform for biological-image analysis. *Nat. Methods* 9, 676–682. doi:10.1038/nmeth.2019.

Semenza, G. L., Rue, E. A., Iyer, N. V., Pang, M. G., and Kearns, W. G. (1996). Assignment of the Hypoxia-Inducible Factor 1α Gene to a Region of Conserved Synteny on Mouse Chromosome 12 and Human Chromosome 14q. *Genomics* 34, 437–439. doi:10.1006/geno.1996.0311.

Taylor, S. E., Bagnall, J., Mason, D., Levy, R., Fernig, D. G., and See, V. (2016). Differential sub-nuclear distribution of hypoxia-inducible factors (HIF)-1 and -2 alpha impacts on their stability and mobility. *Open Biol.* 6, 160195. doi:10.1098/rsob.160195.

Tripathi, S., Pohl, M. O., Zhou, Y., Rodriguez-Frandsen, A., Wang, G., Stein, D. A., et al. (2015). Meta- and Orthogonal Integration of Influenza "OMICs" Data Defines a Role for UBR4 in Virus Budding. *Cell Host Microbe* 18, 723–35. doi:10.1016/j.chom.2015.11.002.

Tyanova, S., Temu, T., and Cox, J. (2016a). The MaxQuant computational platform for mass

spectrometry-based shotgun proteomics. *Nat. Protoc.* 11, 2301–2319. doi:10.1038/nprot.2016.136.

Tyanova, S., Temu, T., Sinitcyn, P., Carlson, A., Hein, M. Y., Geiger, T., et al. (2016b). The Perseus computational platform for comprehensive analysis of (prote)omics data. *Nat. Methods* 13, 731–40. doi:10.1038/nmeth.3901.

Wenger, R. H., and Gassmann, M. (1999). “Molecular Biology of Hypoxia-Inducible Factor-1,” in *Molecular Biology of Hematopoiesis 6*, eds. N. G. Abraham, A. Tabilio, M. Martelli, S. Asano, and A. Donfrancesco, 269–276. doi:10.1007/978-1-4615-4797-6_34.

Wenger, R. H., Rolfs, A., Kvietikova, I., Spielmann, P., Zimmermann, D. R., and Gassmann, M. (1997). The mouse gene for hypoxia-inducible factor-1alpha: genomic organization, expression and characterization of an alternative first exon and 5’ flanking sequence. *Eur. J. Biochem.* 246, 155–165. doi:10.1111/j.1432-1033.1997.t01-1-00155.x.

Wenger, R. H., Rolfs, A., Marti, H. H., Guenet, J. L., and Gassmann, M. (1996). Nucleotide sequence, chromosomal assignment and mRNA expression of mouse hypoxia-inducible factor-1 alpha. *Biochem. Biophys. Res. Commun.* 223, 54–59.

Wenger, R. H., Rolfs, A., Spielmann, P., Zimmermann, D. R., Gassmann, M., and Wenger, B. R. H. (1998). Mouse hypoxia-inducible factor-1alpha is encoded by two different mRNA isoforms: expression from a tissue-specific and a housekeeping-type promoter. *Blood* 91, 3471–80. Available at: http://www.ncbi.nlm.nih.gov/pubmed/9558407.

Wu, D., Potluri, N., Lu, J., Kim, Y., and Rastinejad, F. (2015). Structural integration in hypoxia-inducible factors. *Nature* 524, 303–8. doi:10.1038/nature14883.
